# Supplementary material for: Influence of the Human Field of View on Visual and Non-Visual Quantities in Indoor Environments
Source: Clocks Sleep. 2023 Aug 29;5(3):476–98. doi: 10.3390/clockssleep5030032 (PMC10530223; doi:10.3390/clockssleep5030032)
Supplement: Supplementary file 1 [file clockssleep-05-00032-s001.zip › 05-SI/SI4.html]

 
  

 
 

 

 

 Influence of the human field of view on visual and nonvisual quantities 
 


 
 
 
 
 
 
 
 
 
 


 

 

 

 

 
 
 Influence of the human field of view on visual and nonvisual quantities 
 Supplemental Material: Analysis of spectral measurements 
 


 

     
     Author 
     
              Johannes Zauner, Kai Broszio, Karin Bieske  
           
   
    
  
    
   
  

 

 
 Setup 
 Setup of all the packages and functions. 
 
 
 Code 
       library (tidyverse) 
    library (cowplot) 
    library (gt) 
    library (ggridges) 
    library (patchwork) 
    library (ggtext) 
    library (ggimage) 
    library (ggdist) 
    library (magick) 
    library (readxl) 
    
    #function for creating a void plot. The measurement without the FOV-occluder is  
    #the maximum, with the occluder the minimum. The deep spectral color shows the  
    #reduced part of the plot  
   Red_Plot  <-   function (Data, Bezeichnung_Wellenlaenge, Bezeichnung_Voll,  
                        Bezeichnung_Blende) { 
      #palette  
     regenbogen  <-   c ( rev ( rainbow ( 150 ,  start =   0.4 ,  end =   0.77 )), 
                      rev ( rainbow ( 31 ,  start =   0.20 ,  end =   0.4 )), 
                      rev ( rainbow ( 145 ,  start =   0 ,  end =   0.20 )), 
                      rev ( rainbow ( 75 ,  start =   0.96 ,  end =   1 ))) 
    
      ggplot (Data)  +  
      geom_ridgeline_gradient ( 
        aes ( x= {{ Bezeichnung_Wellenlaenge }},  
            y=  0 ,  
            height= {{ Bezeichnung_Voll }},  
            fill =  {{ Bezeichnung_Wellenlaenge }}),  
        col =   NA )  +  
      geom_ridgeline ( 
        aes ( x= {{ Bezeichnung_Wellenlaenge }},  
            y=  0 ,  
            height=  {{ Bezeichnung_Blende }}),  
        fill =   "white" ,  
        alpha=  0.75 )  +  
      geom_hline ( aes ( yintercept=  0 ),  col =   "white" ) +  
      scale_fill_gradientn ( colors =  regenbogen,  guide =   "none" ) +  
      theme_void () 
   } 
    
    #function to create a void plot. The relative reduction depending on the  
    #wavelength is shown in the spectral color of each wavelength.  
    #A horizontal dashed line shows the median (value is also shown).  
   Red_pct_Plot  <-   function (Data, Bezeichnung_Wellenlaenge, Bezeichnung_Voll,  
                            Bezeichnung_Blende) { 
      #palette  
     regenbogen  <-   c ( rev ( rainbow ( 150 ,  start =   0.4 ,  end =   0.77 )), 
                      rev ( rainbow ( 31 ,  start =   0.20 ,  end =   0.4 )), 
                      rev ( rainbow ( 145 ,  start =   0 ,  end =   0.20 )), 
                      rev ( rainbow ( 75 ,  start =   0.96 ,  end =   1 ))) 
    
     Data_int  <-  Data  %>%   
        mutate ( PCT =  {{ Bezeichnung_Blende }}  /  {{ Bezeichnung_Voll }}, 
               PCT =   ifelse (PCT  >   1 ,  NA , PCT)) 
    
      ggplot (Data_int)  +  
        geom_line ( aes ( y =  PCT,  
                      x =  {{ Bezeichnung_Wellenlaenge }},  
                      col =  {{ Bezeichnung_Wellenlaenge }}), 
                  linewidth =   1 , 
                  na.rm =   TRUE )  +  
        coord_cartesian ( ylim =   c ( 0 ,  1 ),  xlim =   c ( 380 ,  780 ),  clip =   "off" )  +  
        geom_hline ( aes ( yintercept =   median ((PCT),  na.rm =   TRUE )), 
                   col =   "black" , 
                   lty =   2 )  +  
        geom_label ( aes ( label =  scales ::  percent ( median ((PCT),  na.rm =   TRUE )),  
                       x =   580 ,  y =   median ((PCT),  na.rm =   TRUE )),  
                   fill =   "white" ,  label.size =   NA ,  
                   label.padding =   unit ( 0.05 ,  "lines" ),  
                   hjust =   0.5 ) +  
        scale_color_gradientn ( colors =  regenbogen,  guide =   "none" )  +  
        scale_y_continuous ( breaks =   c ( 0 ,  1 ), 
                           labels =  scales :: percent) +  
        theme_void () +  
        theme ( axis.text.y =   element_text (), 
              axis.line.y =   element_line ()) 
   } 
    
    #Function that brings both plots together  
   Combined_Plot  <-   function (...,  Aufteilung =   c ( 0.75 ,  0.25 ),  
                              Ordner =   "" , Bezeichnung) { 
     Plot  <-   Red_Plot (...)  /   Red_pct_Plot (...)  +  
        plot_layout ( heights =  Aufteilung) 
    
     Plot 
      ggsave ( paste0 (Ordner, Bezeichnung,  ".png" ), Plot,  width =   2.5 ,  height =   2 ) 
      
   } 
    
    #takes a filename and a path and fits it together to a full pathname  
   image_file_path  <-   function (Bildname, Pfad) { 
      if_else (Bildname  ==   "" ,  "&mdash;" ,  paste0 (Pfad, "/" ,Bildname)) 
   } 
    
    #creates a table  
   Table  <-   function (Daten, Titel) { 
     Table  <-  Daten  %>%   
      mutate ( 
        MDER_Voll =  MEDI_Voll / E_Voll, 
        MDER_Blende =  MEDI_Blende / E_Blende, 
        MDER_pct =  scales ::  percent ( -  1  +  round (MDER_Blende / MDER_Voll,  2 )) 
     )  %>%   
      gt ()  %>%   
      tab_header ( title =  Titel)  %>%  
      fmt_number ( columns =   starts_with ( c ( "E_" ,  "MEDI_" ))  &  is.numeric,  
                 decimals=  0 )  %>%  
      fmt_number ( columns =   starts_with ( c ( "MDER_" ))  &  is.numeric,  
                 decimals=  3 )  %>%  
      cols_move (Plot, Bild)  %>%  
      cols_merge ( c (MEDI_Voll, MEDI_Blende, MEDI_pct), 
                 pattern =   "<i>2π:</i> {1} lx<i style = 'color:white'>a</i><br><i>FOV:</i> {2} lx<i style = 'color:white'>a</i><br><strong>{3}</strong><i style = 'color:white'>a</i>" )  %>%  
      cols_merge ( c (E_Voll, E_Blende, E_pct), 
                 pattern =   "<i>2π:</i> {1} lx<i style = 'color:white'>a</i><br><i>FOV:</i> {2} lx<i style = 'color:white'>a</i><br><strong>{3}</strong><i style = 'color:white'>a</i>" )  %>%  
      cols_merge ( c (MDER_Voll, MDER_Blende, MDER_pct), 
      pattern =   "<i>2π: </i> {1}<i style = 'color:white'>a</i><br><i>FOV:</i> {2}<i style = 'color:white'>a</i><br><strong>{3}</strong><i style = 'color:white'>a</i>" )  %>%  
      cols_label ( 
        Szene =   "Name" , 
        Plot =   "rSPD" , 
        E_Voll =   "Illuminance" , 
        MEDI_Voll =   "MEDI" , 
        Bild =   "" , 
        MDER_Voll =   "MDER"  
     )  %>%  
      cols_align ( align =   "center" )  %>%  
      text_transform ( 
        locations =   cells_body ( columns =  Bild,  rows =  Bild  !=   "&mdash;" ), 
        fn =   function (x) { 
          local_image ( filename =  x,  height =   100 ) 
         } 
       )  %>%  
      text_transform ( 
        locations =   cells_body ( columns =  Plot), 
        fn =   function (x) { 
          local_image ( filename =  x,  height =   100 ) 
         } 
     )  %>%  
      fmt_markdown ( columns =  Bild,  rows =  Bild  ==   "&mdash;" ) 
    
     Table 
   } 
    
    #add names of columns with FOV-occluders  
   Blendennamen  <-   function (Bezeichnung) { 
      str_c ( "Ev_SK_" ,  str_sub (Bezeichnung,  start =   4 )) 
   } 
    
    #New Function to identify the Scene with the FOV-occluder  
   Blendennamen2  <-   function (Bezeichnung) { 
      str_c (Bezeichnung,  "_SK" ) 
   } 
    
    #create an empty tibble to fit all the measurements into  
   Meta  <-   tibble ()        
 
 
 
 
 Homeoffice 
 
 
 Code 
       #Setting up the Information and Paths  
   Bezeichnungen  <-   c ( "Ev_Morgen" ,  "Ev_Tag" ,  "Ev_Abend" ,  "Ev_Nacht" ) 
   Wellenlängenspalte  <-   "Name"  
   Pfad_Bild  <-   "Pictures/Homeoffice"  
   Pfad_Spec  <-   "Results/Homeoffice"  
   file_homeoffice  <-   "Data/Homeoffice/Heimarbeitsplatz.csv"  
   file_homeoffice_params  <-   "Data/Homeoffice/Homeoffice_Parameter.csv"  
    
    #Import the spectral Data    
   Homeoffice  <-   
      read.csv (file_homeoffice,  sep =   ";" ,  dec =   "," ,  skip =   3 )  %>%   
      slice ( - ( 1  :  126 ))  %>%   
      slice ( 1  :  401 )  %>%   
      mutate ( across ( .fns=  function (x)  str_replace (x,  "," ,  "." )), 
             across ( .fns= as.numeric), 
             Name =   as.integer (Name)) 
    
    #Import the visual and nonvisual quantities  
   Homeoffice_params  <-   read.csv (file_homeoffice_params,  sep =   ";" )  %>%   
      mutate ( Bild =   image_file_path (Bild, Pfad_Bild), 
             Plot =   image_file_path ( str_c (Plot,  ".png" ), Pfad_Spec), 
             E_pct =  scales ::  percent ( -  1  +  round (E_Blende / E_Voll,  2 )), 
             MEDI_pct =  scales ::  percent ( -  1  +  round (MEDI_Blende / MEDI_Voll,  2 )) 
     ) 
   Homeoffice_params  <-  Homeoffice_params  %>%   mutate ( MEDI_pct =  MEDI_pct  %>%   
                                                        str_replace ( "  \\  .0%" ,  "  \\  %" )) 
    
    #Generating the Arguments for the spectral plots  
   Homeoffice_args  <-  
      list ( 
        Data =   list (Homeoffice), 
        Bezeichnung_Wellenlaenge =   syms (Wellenlängenspalte), 
        Bezeichnung_Voll =   syms (Bezeichnungen), 
        Bezeichnung_Blende =   syms ( Blendennamen (Bezeichnungen)), 
        Ordner =   list ( "Results/Homeoffice/" ), 
        Bezeichnung =   as.list (Bezeichnungen) 
     ) 
    
    #Generating the spectral plots  
    pwalk (Homeoffice_args,Combined_Plot) 
    
    #Changing Names to english  
   Homeoffice_params  <-  Homeoffice_params  %>%   
      mutate ( 
        Szene =   case_match (Szene, 
          "Morgen"   ~   "Morning" , 
          "Tag"   ~   "Daytime" , 
          "Abend"   ~   "Evening" , 
          "Nacht"   ~   "Night"  
       ) 
     ) 
    
    #Generating the table, displaying and saving it  
   Table_Homeoffice  <-   Table (Homeoffice_params,  
                              NULL )  
    
   Table_Homeoffice        
 
 

 
 
 
   
    
     
        
       rSPD 
       Name 
       Illuminance 
       MEDI 
       MDER 
     
   
   
        
   
 Morning 
  2π:  450 lx a   FOV:  184 lx a   -59%  a  
  2π:  426 lx a   FOV:  171 lx a   -60%  a  
  2π:   0.947 a   FOV:  0.927 a   -2%  a   
        
   
 Daytime 
  2π:  390 lx a   FOV:  161 lx a   -59%  a  
  2π:  324 lx a   FOV:  131 lx a   -60%  a  
  2π:   0.831 a   FOV:  0.812 a   -2%  a   
        
   
 Evening 
  2π:  199 lx a   FOV:  83 lx a   -58%  a  
  2π:  100 lx a   FOV:  42 lx a   -58%  a  
  2π:   0.502 a   FOV:  0.501 a   0%  a   
        
   
 Night 
  2π:  139 lx a   FOV:  58 lx a   -58%  a  
  2π:  55 lx a   FOV:  23 lx a   -59%  a  
  2π:   0.397 a   FOV:  0.396 a   0%  a   
   
  
  
 
 
 
 
 Code 
       gtsave (Table_Homeoffice,  "Results/Table_Homeoffice.png" ,  zoom =   4 ) 
    
    #Putting data to the Meta-Frame  
   Meta  <-   rbind (Meta,  
                  cbind ( Projekt =   "Homeoffice" , Homeoffice_params))        
 
 
 
 
 Learning Room 
  Link to the Project Publication  
 
 
 Code 
       #Setting up the Information and Paths  
   Pfad_Bild  <-   "Pictures/Lernraum"  
   Pfad_Spec  <-   "Results/Lernraum"  
   Pfad  <-   "Data/Lernraum"  
   Wellenlängenspalte  <-   "Wavelength"  
   Bezeichnungen  <-   c ( "Ev_Abend" ,  "Ev_Tag" ,  "Ev_Morgen" ) 
   file_lernraum_params  <-   "Data/Lernraum/Lernraum_Parameter.csv"  
   file_lernraum  <-   list.files (Pfad,  pattern =   "[.]csv$" ) 
   filepath_lernraum  <-  
      list.files (Pfad,  pattern =   "Ev_.+[.]csv$" ,  full.names =   TRUE )  %>%   
      set_names ( c (Bezeichnungen,  Blendennamen (Bezeichnungen))) 
    
    #Import the spectral Data  
   Lernraum  <-    map_dfr (filepath_lernraum, read.csv,  skip =   11 ,  .id =   "Quelle" )  %>%   
        pivot_wider ( names_from =  Quelle,  
                    values_from =  Spectral.Irradiance..W..sqm.nm..) 
    
    #Import the visual and nonvisual quantities  
   Lernraum_params  <-   read.csv (file_lernraum_params,  sep =   ";" )  %>%   
      mutate ( Bild =   image_file_path (Bild, Pfad_Bild), 
             Plot =   image_file_path ( str_c (Plot,  ".png" ), Pfad_Spec), 
             E_pct =  scales ::  percent ( -  1  +  round (E_Blende / E_Voll,  2 )), 
             MEDI_pct =  scales ::  percent ( -  1  +  round (MEDI_Blende / MEDI_Voll,  2 )) 
     ) 
    
    #Generating the Arguments for the spectral plots  
   Lernraum_args  <-  
      list ( 
        Data =   list (Lernraum), 
        Bezeichnung_Wellenlaenge =   syms (Wellenlängenspalte), 
        Bezeichnung_Voll =   syms (Bezeichnungen), 
        Bezeichnung_Blende =   syms ( Blendennamen (Bezeichnungen)), 
        Ordner =   list ( paste0 (Pfad_Spec,  "/" )), 
        Bezeichnung =   as.list (Bezeichnungen) 
     ) 
    
    #Generating the spectral plots  
    pwalk (Lernraum_args,Combined_Plot) 
    
    #Changing Names to english  
   Lernraum_params  <-  Lernraum_params  %>%   
      mutate ( 
        Szene =   case_match (Szene, 
          "Morgen"   ~   "Morning" , 
          "Tag"   ~   "Daytime" , 
          "Abend"   ~   "Evening" , 
          "Nacht"   ~   "Night"  
       ) 
     ) 
    
    #Generating the table, displaying and saving it  
   Table_Lernraum  <-   Table (Lernraum_params,  NULL ) 
    
   Table_Lernraum        
 
 

 
 
 
   
    
     
        
       rSPD 
       Name 
       Illuminance 
       MEDI 
       MDER 
     
   
   
        
   
 Morning 
  2π:  261 lx a   FOV:  232 lx a   -11%  a  
  2π:  242 lx a   FOV:  215 lx a   -11%  a  
  2π:   0.927 a   FOV:  0.927 a   0%  a   
        
   
 Daytime 
  2π:  209 lx a   FOV:  164 lx a   -22%  a  
  2π:  128 lx a   FOV:  100 lx a   -22%  a  
  2π:   0.612 a   FOV:  0.610 a   0%  a   
        
   
 Evening 
  2π:  147 lx a   FOV:  87 lx a   -41%  a  
  2π:  54 lx a   FOV:  31 lx a   -43%  a  
  2π:   0.367 a   FOV:  0.356 a   -3%  a   
   
  
  
 
 
 
 
 Code 
       gtsave (Table_Lernraum,  "Results/Table_Lernraum.png" ,  zoom =   4 ) 
    
    #Putting data to the Meta-Frame  
   Meta  <-   rbind (Meta,  
                  cbind ( Projekt =   "Lernraum" , Lernraum_params))        
 
 
 
 
 Industry Workplace (Machine) 
  Link to the Project Publication  
 
 
 Code 
       #Setting up the Information and Paths  
   Pfad_Bild  <-   "Pictures/RHI/Maschinenarbeitsplatz"  
   Pfad_Spec  <-   "Results/RHI/Maschinenarbeitsplatz"  
   Wellenlängenspalte  <-   "Wavelength"  
   Bezeichnungen  <-   c ( "Ev_Morgen" ,  "Ev_Tag" ,  "Ev_Nacht" ,  "Ev_Nachtende" ) 
   file_RHI_params  <-   "Data/RHI/Maschinenarbeitsplatz/RHI_Parameter.csv"  
   file_RHI  <-   "Data/RHI/Maschinenarbeitsplatz/AP1-3.csv"  
    
    #Import the spectral Data  
   RHI  <-   read.csv (file_RHI,  sep =   ";" ,  dec =   "," ,  skip =   16 ,  header =   FALSE )  %>%   
      select (V1, V6, V9, V13, V14, V7, V8, V11, V15)  %>%   
      set_names ( c (Wellenlängenspalte, Bezeichnungen,  Blendennamen (Bezeichnungen))) 
    
    #Import the visual and nonvisual quantities  
   RHI_params  <-   read.csv (file_RHI_params,  sep =   ";" )  %>%  
      rowwise ()  %>%   
      mutate ( Bild =   image_file_path (Bild, Pfad_Bild), 
             Plot =   image_file_path ( str_c (Plot,  ".png" ), Pfad_Spec), 
             E_pct =  scales ::  percent ( -  1  +  round (E_Blende / E_Voll,  2 )), 
             MEDI_pct =  scales ::  percent ( -  1  +  round (MEDI_Blende / MEDI_Voll,  2 )) 
     ) 
    
    #Generating the Arguments for the spectral plots  
   RHI_args  <-  
      list ( 
        Data =   list (RHI), 
        Bezeichnung_Wellenlaenge =   syms (Wellenlängenspalte), 
        Bezeichnung_Voll =   syms (Bezeichnungen), 
        Bezeichnung_Blende =   syms ( Blendennamen (Bezeichnungen)), 
        Ordner =   list ( paste0 (Pfad_Spec,  "/" )), 
        Bezeichnung =   as.list (Bezeichnungen) 
     ) 
    
    #Changing Names to english  
   RHI_params  <-  RHI_params  %>%   
      mutate ( 
        Szene =   case_match (Szene, 
          "Morgen"   ~   "Morning" , 
          "Tag"   ~   "Daytime" , 
          "Abend"   ~   "Evening" , 
          "Nacht"   ~   "Night" , 
          "Nachtende"   ~   "Night's End"  
       ) 
     ) 
    
    #Generating the spectral plots  
    pwalk (RHI_args,Combined_Plot) 
    
    #Generating the table, displaying and saving it  
   Table_RHI_Maschine  <-   Table (RHI_params,  NULL ) 
    
   Table_RHI_Maschine        
 
 

 
 
 
   
    
     
        
       rSPD 
       Name 
       Illuminance 
       MEDI 
       MDER 
     
   
   
        
   
 Morning 
  2π:  449 lx a   FOV:  433 lx a   -3%  a  
  2π:  412 lx a   FOV:  399 lx a   -3%  a  
  2π:   0.918 a   FOV:  0.920 a   0%  a   
        
   
 Daytime 
  2π:  344 lx a   FOV:  331 lx a   -4%  a  
  2π:  278 lx a   FOV:  268 lx a   -4%  a  
  2π:   0.809 a   FOV:  0.809 a   0%  a   
        
   
 Night 
  2π:  156 lx a   FOV:  150 lx a   -4%  a  
  2π:  72 lx a   FOV:  70 lx a   -3%  a  
  2π:   0.463 a   FOV:  0.466 a   1%  a   
        
   
 Night's End 
  2π:  218 lx a   FOV:  211 lx a   -3%  a  
  2π:  100 lx a   FOV:  96 lx a   -3%  a  
  2π:   0.457 a   FOV:  0.457 a   0%  a   
   
  
  
 
 
 
 
 Code 
       gtsave (Table_RHI_Maschine,  "Results/Table_RHI_Maschine.png" ,  zoom =   4 ) 
    
    #Putting data to the Meta-Frame  
   Meta  <-   rbind (Meta,  
                  cbind ( Projekt =   "Industriehalle" , RHI_params))        
 
 
 
 
 Industry Workplace (Packaging) 
  Link to the Project Publication  
 
 
 Code 
       #Setting up the Information and Paths  
   Pfad_Bild  <-   "Pictures/RHI/Verpackung"  
   Pfad_Spec  <-   "Results/RHI/Verpackung"  
   Wellenlängenspalte  <-   "Wavelength"  
   Bezeichnungen  <-   c ( "Ev_Morgen" ,  "Ev_Tag" ,  "Ev_Nacht" ,  "Ev_Nachtende" ) 
   file_RHI2_params  <-   "Data/RHI/Verpackung/RHI_Parameter.csv"  
   file_RHI2  <-   "Data/RHI/Verpackung/AP2.csv"  
    
    #Import the spectral Data  
   RHI2  <-   read.csv (file_RHI2,  sep =   ";" ,  dec =   "," ,  skip =   16 ,  header =   FALSE )  %>%   
      select (V1, V9, V4, V7, V10, V3, V5, V6, V11)  %>%   
      set_names ( c (Wellenlängenspalte, Bezeichnungen,  Blendennamen (Bezeichnungen))) 
    
    #Import the visual and nonvisual quantities  
   RHI2_params  <-   read.csv (file_RHI2_params,  sep =   ";" )  %>%  
      rowwise ()  %>%   
      mutate ( Bild =   image_file_path (Bild, Pfad_Bild), 
             Plot =   image_file_path ( str_c (Plot,  ".png" ), Pfad_Spec), 
             E_pct =  scales ::  percent ( -  1  +  round (E_Blende / E_Voll,  2 )), 
             MEDI_pct =  scales ::  percent ( -  1  +  round (MEDI_Blende / MEDI_Voll,  2 )) 
     ) 
    
    #Generating the Arguments for the spectral plots  
   RHI2_args  <-  
      list ( 
        Data =   list (RHI2), 
        Bezeichnung_Wellenlaenge =   syms (Wellenlängenspalte), 
        Bezeichnung_Voll =   syms (Bezeichnungen), 
        Bezeichnung_Blende =   syms ( Blendennamen (Bezeichnungen)), 
        Ordner =   list ( paste0 (Pfad_Spec,  "/" )), 
        Bezeichnung =   as.list (Bezeichnungen) 
     ) 
    
    #Changing Names to english  
   RHI2_params  <-  RHI2_params  %>%   
      mutate ( 
        Szene =   case_match (Szene, 
          "Morgen"   ~   "Morning" , 
          "Tag"   ~   "Daytime" , 
          "Abend"   ~   "Evening" , 
          "Nacht"   ~   "Night" , 
          "Nachtende"   ~   "Night's End"  
       ) 
     ) 
    
    #Generating the spectral plots  
    pwalk (RHI2_args,Combined_Plot) 
    
    #Generating the table, displaying and saving it  
   Table_RHI_Verpackung  <-   Table (RHI2_params,  NULL ) 
    
   Table_RHI_Verpackung        
 
 

 
 
 
   
    
     
        
       rSPD 
       Name 
       Illuminance 
       MEDI 
       MDER 
     
   
   
        
   
 Morning 
  2π:  460 lx a   FOV:  206 lx a   -55%  a  
  2π:  322 lx a   FOV:  151 lx a   -53%  a  
  2π:   0.702 a   FOV:  0.736 a   5%  a   
        
   
 Daytime 
  2π:  225 lx a   FOV:  154 lx a   -31%  a  
  2π:  158 lx a   FOV:  110 lx a   -31%  a  
  2π:   0.704 a   FOV:  0.712 a   1%  a   
        
   
 Night 
  2π:  153 lx a   FOV:  120 lx a   -22%  a  
  2π:  97 lx a   FOV:  79 lx a   -19%  a  
  2π:   0.633 a   FOV:  0.654 a   3%  a   
        
   
 Night's End 
  2π:  199 lx a   FOV:  128 lx a   -36%  a  
  2π:  122 lx a   FOV:  81 lx a   -33%  a  
  2π:   0.614 a   FOV:  0.637 a   4%  a   
   
  
  
 
 
 
 
 Code 
       gtsave (Table_RHI_Verpackung,  "Results/Table_RHI_Verpackung.png" ,  zoom =   4 ) 
    
    #Putting data to the Meta-Frame  
   Meta  <-   rbind (Meta, 
                  cbind ( Projekt =   "Industriehalle2" , RHI2_params))        
 
 
 
 
 BAuA Lab-Study (tilted downwards) 
 
 
 Code 
       #Setting up the Information and Paths  
   Pfad  <-   "Data/BAuA/geneigt"  
   Pfad_Results  <-   "Results/BAuA/geneigt"  
   Wellenlängenspalte  <-   "Name"  
   file_params  <-   paste0 (Pfad,  "/Parameter.csv" ) 
   file_Spektrum  <-   paste0 (Pfad,  "/BAuA-Studie_Messungen.csv" ) 
    
    #Import the spectral Data  
   Spektraldaten  <-   read.csv (file_Spektrum,  sep =   ";" ,  dec =   "," )  %>%   
      select ( 1  :  10 ) 
    
    #Import the visual and nonvisual quantities  
   Params  <-   read.csv (file_params,  sep =   ";" ,  dec =   "," )  %>%  
      select ( 1  :  4 )  %>%   filter ( !  str_detect (Name,  "Mun" ))  %>%   
      separate (Name,  c ( "Name" ,  "SK" ),  sep =   "_" )  %>%   
      mutate ( SK =   recode (SK,  "SK"   =   "Blende" , 
                         .missing =   "Voll" ))  %>%   
      pivot_wider ( values_from =   c (E,MEDI, CCT),  names_from =  SK)  %>%   
      mutate ( Bild =   image_file_path ( "" , Pfad), 
             Plot =   
               image_file_path ( str_c (Name,  ".png" )  %>%   
                                 str_replace ( " " ,  "." ), Pfad_Results), 
             E_pct =  scales ::  percent ( -  1  +  round (E_Blende / E_Voll,  2 )), 
             MEDI_pct =  scales ::  percent ( -  1  +  round (MEDI_Blende / MEDI_Voll,  2 )) 
     )  %>%   
      select ( - CCT_Voll,  - CCT_Blende)  %>%   
      relocate (Bild, Plot)  %>%   
      rename ( "Szene"   =   "Name" ) 
    
    #Adjust Scene-Names  
   Bezeichnungen  <-  Params $ Szene  %>%   str_replace ( " " ,  "." ) 
    
    #Generating the Arguments for the spectral plots  
   Argumente  <-  
      list ( 
        Data =   list (Spektraldaten), 
        Bezeichnung_Wellenlaenge =   syms (Wellenlängenspalte), 
        Bezeichnung_Voll =   syms (Bezeichnungen), 
        Bezeichnung_Blende =   syms ( Blendennamen2 (Bezeichnungen)), 
        Ordner =   list ( paste0 (Pfad_Results,  "/" )), 
        Bezeichnung =   as.list (Bezeichnungen) 
     ) 
    
    #Generating the spectral plots  
    pwalk (Argumente,Combined_Plot) 
    
    #Generating the table, displaying and saving it  
   Tabellendaten  <-   
    Table (Params,  NULL )  %>%   cols_hide (Bild) 
    
   Tabellendaten        
 
 

 
 
 
   
    
     
       rSPD 
       Name 
       Illuminance 
       MEDI 
       MDER 
     
   
   
        
 warm low 
  2π:  208 lx a   FOV:  168 lx a   -19%  a  
  2π:  99 lx a   FOV:  80 lx a   -20%  a  
  2π:   0.477 a   FOV:  0.475 a   0%  a   
        
 cold low 
  2π:  209 lx a   FOV:  168 lx a   -19%  a  
  2π:  209 lx a   FOV:  168 lx a   -20%  a  
  2π:   1.000 a   FOV:  0.995 a   0%  a   
        
 cold bright 
  2π:  579 lx a   FOV:  466 lx a   -20%  a  
  2π:  581 lx a   FOV:  465 lx a   -20%  a  
  2π:   1.002 a   FOV:  0.998 a   0%  a   
   
  
  
 
 
 
 
 Code 
       gtsave (Tabellendaten,  "Results/Table_BAuA-Studie_geneigt.png" ,  zoom =   4 ) 
    
    #Putting data to the Meta-Frame  
   Meta  <-   rbind (Meta, 
                  cbind ( Projekt =   "BAuA Studie" , Params))        
 
 
 
 
 BAuA Lab-Study (vertical) 
 
 
 Code 
       #Setting up the Information and Paths  
   Pfad  <-   "Data/BAuA/vertikal"  
   Pfad_Bild  <-   "Pictures/BAuA/vertikal"  
   Pfad_Results  <-   "Results/BAuA/vertikal"  
   Wellenlängenspalte  <-   "Name"  
   file_params  <-   paste0 (Pfad,  "/Parameter.csv" ) 
   file_Spektrum  <-   paste0 (Pfad,  "/Messdaten.csv" ) 
    
    #Import the spectral Data  
   Spektraldaten  <-   read.csv (file_Spektrum,  sep =   ";" ,  dec =   "," )  %>%   
      select ( 1  :  10 ) 
    
    #Import the visual and nonvisual quantities  
   Params  <-   read.csv (file_params,  sep =   ";" ,  dec =   "," )  %>%  
      select ( 1  :  5 )  %>%   filter ( !  str_detect (Name,  "Mun" ))  %>%   
      separate (Name,  c ( "Name" ,  "SK" ),  sep =   "_" )  %>%   
      mutate ( SK =   recode (SK,  "SK"   =   "Blende" , 
                         .missing =   "Voll" ))  %>%   
      pivot_wider ( values_from =   c (E,MEDI, CCT),  names_from =  SK)  %>%   
      rowwise ()  %>%   
      mutate ( Bild =   image_file_path (Bild, Pfad_Bild), 
             Plot =   
               image_file_path ( str_c (Name,  ".png" )  %>%   
                                 str_replace ( " " ,  "." ), Pfad_Results), 
             E_pct =  scales ::  percent ( -  1  +  round (E_Blende / E_Voll,  2 )), 
             MEDI_pct =  scales ::  percent ( -  1  +  round (MEDI_Blende / MEDI_Voll,  2 )) 
     )  %>%   
      select ( - CCT_Voll,  - CCT_Blende)  %>%   
      relocate (Bild, Plot)  %>%   
      rename ( "Szene"   =   "Name" ) %>%   
      arrange (Szene) 
    
    #Adjust Scene-Names  
   Bezeichnungen  <-  Params $ Szene  %>%   str_replace ( " " ,  "." ) 
    
    #Generating the Arguments for the spectral plots  
   Argumente  <-  
      list ( 
        Data =   list (Spektraldaten), 
        Bezeichnung_Wellenlaenge =   syms (Wellenlängenspalte), 
        Bezeichnung_Voll =   syms (Bezeichnungen), 
        Bezeichnung_Blende =   syms ( Blendennamen2 (Bezeichnungen)), 
        Ordner =   list ( paste0 (Pfad_Results,  "/" )), 
        Bezeichnung =   as.list (Bezeichnungen) 
     ) 
    
    #Generating the spectral plots  
    pwalk (Argumente,Combined_Plot) 
    
    #Generating the table, displaying and saving it  
   Tabellendaten  <-   
    Table (Params  %>%   arrange ( rev (Szene)),  NULL ) 
    
   Tabellendaten        
 
 

 
 
 
   
    
     
        
       rSPD 
       Name 
       Illuminance 
       MEDI 
       MDER 
     
   
   
        
   
 warm low 
  2π:  296 lx a   FOV:  212 lx a   -28%  a  
  2π:  141 lx a   FOV:  101 lx a   -28%  a  
  2π:   0.476 a   FOV:  0.476 a   0%  a   
        
   
 cold low 
  2π:  298 lx a   FOV:  213 lx a   -29%  a  
  2π:  300 lx a   FOV:  214 lx a   -29%  a  
  2π:   1.007 a   FOV:  1.005 a   0%  a   
        
   
 cold bright 
  2π:  830 lx a   FOV:  592 lx a   -29%  a  
  2π:  838 lx a   FOV:  595 lx a   -29%  a  
  2π:   1.010 a   FOV:  1.005 a   0%  a   
   
  
  
 
 
 
 
 Code 
       gtsave (Tabellendaten,  "Results/Table_BAuA-Studie_vertikal.png" ,  zoom =   4 ) 
    
    #Putting data to the Meta-Frame  
   Meta  <-   rbind (Meta,  
                  cbind ( Projekt =   "BAuA Studie" , Params))        
 
 
 
 
 TUB-HK Lab-Study 
 
 
 Code 
       #Setting up the Information and Paths  
   Pfad  <-   "Data/TUB-HK"  
   Pfad_Bild  <-   "Pictures/TUB-HK"  
   Pfad_Spec  <-   "Results/TUB-HK"  
   Wellenlängenspalte  <-   "Name"  
   file_params  <-   paste0 (Pfad,  "/Parameter.csv" ) 
   file_Spektrum  <-   paste0 (Pfad,  "/Messdaten.csv" ) 
    
    #Import the spectral Data  
   Spektraldaten  <-   read.csv (file_Spektrum,  sep =   ";" ,  dec =   "," )  %>%   
      select ( 1  :  4 ) 
    
    #Import the visual and nonvisual quantities  
   Params  <-   read.csv (file_params,  sep =   ";" ,  dec =   "," )  %>%  
      select ( 1  :  5 )  %>%   filter ( !  str_detect (Name,  "Mun" ))  %>%   
      separate (Name,  c ( "Name" ,  "SK" ),  sep =   "_" )  %>%   
      mutate ( SK =   recode (SK,  "SK"   =   "Blende" , 
                         .missing =   "Voll" ))  %>%   
      pivot_wider ( values_from =   c (E,MEDI, CCT),  names_from =  SK)  %>%   
      rowwise ()  %>%   
      mutate ( Bild =   image_file_path (Bild, Pfad_Bild), 
             Plot =   
               image_file_path ( str_c (Name,  ".png" )  %>%   
                                 str_replace ( " " ,  "." ), Pfad_Spec), 
             E_pct =  scales ::  percent ( -  1  +  round (E_Blende / E_Voll,  2 )), 
             MEDI_pct =  scales ::  percent ( -  1  +  round (MEDI_Blende / MEDI_Voll,  2 )) 
     )  %>%   
      select ( - CCT_Voll,  - CCT_Blende)  %>%   
      relocate (Bild, Plot)  %>%   
      rename ( "Szene"   =   "Name" ) 
    
    #Changing Names to english  
   Params  <-  Params  %>%   
      mutate ( 
        Szene =   case_match (Szene, 
          "Halbkugel"   ~   "Halfdome"  
       ) 
     ) 
    
    #Adjust Scene-Names  
   Bezeichnungen  <-  Params $ Szene  %>%   str_replace ( " " ,  "." ) 
    
    #Generating the Arguments for the spectral plots  
   Argumente  <-  
      list ( 
        Data =   list (Spektraldaten), 
        Bezeichnung_Wellenlaenge =   syms (Wellenlängenspalte), 
        Bezeichnung_Voll =   syms ( "Ulbrichtkugel" ), 
        Bezeichnung_Blende =   syms ( Blendennamen2 ( "Ulbrichtkugel" )), 
        Ordner =   list ( paste0 (Pfad_Spec,  "/" )), 
        Bezeichnung =   as.list (Bezeichnungen) 
     ) 
    
    #Generating the spectral plots  
    pwalk (Argumente,Combined_Plot) 
    
    #Generating the table, displaying and saving it  
   Tabellendaten  <-   
    Table (Params, NULL ) 
    
   Tabellendaten        
 
 

 
 
 
   
    
     
        
       rSPD 
       Name 
       Illuminance 
       MEDI 
       MDER 
     
   
   
        
   
 Halfdome 
  2π:  1,982 lx a   FOV:  1,829 lx a   -8%  a  
  2π:  2,323 lx a   FOV:  2,147 lx a   -8%  a  
  2π:   1.172 a   FOV:  1.174 a   0%  a   
   
  
  
 
 
 
 
 Code 
       gtsave (Tabellendaten,  "Results/Table_TUB_HK_Studie.png" ,  zoom =   4 ) 
    
    #Putting data to the Meta-Frame  
   Meta  <-   rbind (Meta, 
                  cbind ( Projekt =   "TUB-Halbk. Studie" , Params))        
 
 
 
 
 TUB-BAuA Lab-Study (tilted downwards) 
 
 
 Code 
       #Setting up the Information and Paths  
   Pfad  <-   "Data/TUB-BAuA/geneigt"  
   Pfad_Spec  <-   "Results/TUB-BAuA/geneigt"  
   Wellenlängenspalte  <-   "Name"  
   file_params  <-   paste0 (Pfad,  "/Parameter.csv" ) 
   file_Spektrum  <-   paste0 (Pfad,  "/Messdaten.csv" ) 
    
    #Import the spectral Data  
   Spektraldaten  <-   read.csv (file_Spektrum,  sep =   ";" ,  dec =   "," )  %>%   
      select ( 1  :  13 ) 
    
    #Import the visual and nonvisual quantities  
   Params  <-   read.csv (file_params,  sep =   ";" ,  dec =   "," )  %>%  
      select ( 1  :  4 )  %>%   filter ( !  str_detect (Name,  "Mun" ))  %>%   
      separate (Name,  c ( "Name" ,  "SK" ),  sep =   "_" )  %>%   
      mutate ( SK =   recode (SK,  "SK"   =   "Blende" , 
                         .missing =   "Voll" ))  %>%   
      pivot_wider ( values_from =   c (E,MEDI, CCT),  names_from =  SK)  %>%   
      mutate ( Bild =   image_file_path ( "" , Pfad), 
             Plot =   
               image_file_path ( str_c (Name,  ".png" )  %>%   
                                 str_replace ( " " ,  "." ), Pfad_Spec), 
             E_pct =  scales ::  percent ( -  1  +  round (E_Blende / E_Voll,  2 )), 
             MEDI_pct =  scales ::  percent ( -  1  +  round (MEDI_Blende / MEDI_Voll,  2 )) 
     )  %>%   
      select ( - CCT_Voll,  - CCT_Blende)  %>%   
      relocate (Bild, Plot)  %>%   
      rename ( "Szene"   =   "Name" ) 
    
    #Adjust Scene-Names  
   Bezeichnungen  <-  Params $ Szene  %>%   str_replace ( " " ,  "." ) 
    
    #Generating the Arguments for the spectral plots  
   Argumente  <-  
      list ( 
        Data =   list (Spektraldaten), 
        Bezeichnung_Wellenlaenge =   syms (Wellenlängenspalte), 
        Bezeichnung_Voll =   syms (Bezeichnungen), 
        Bezeichnung_Blende =   syms ( Blendennamen2 (Bezeichnungen)), 
        Ordner =   list ( paste0 (Pfad_Spec,  "/" )), 
        Bezeichnung =   as.list (Bezeichnungen) 
     ) 
    
    #Generating the spectral plots  
    pwalk (Argumente,Combined_Plot) 
    
    #Generating the table, displaying and saving it  
   Tabellendaten  <-   
    Table (Params,  NULL )  %>%   cols_hide (Bild) 
    
   Tabellendaten        
 
 

 
 
 
   
    
     
       rSPD 
       Name 
       Illuminance 
       MEDI 
       MDER 
     
   
   
        
 LS1 
  2π:  236 lx a   FOV:  214 lx a   -10%  a  
  2π:  106 lx a   FOV:  97 lx a   -9%  a  
  2π:   0.450 a   FOV:  0.454 a   1%  a   
        
 LS2 
  2π:  146 lx a   FOV:  133 lx a   -9%  a  
  2π:  222 lx a   FOV:  199 lx a   -11%  a  
  2π:   1.515 a   FOV:  1.495 a   -1%  a   
        
 LS3 
  2π:  138 lx a   FOV:  126 lx a   -9%  a  
  2π:  196 lx a   FOV:  181 lx a   -7%  a  
  2π:   1.419 a   FOV:  1.444 a   2%  a   
        
 DIM 
  2π:  8 lx a   FOV:  8 lx a   -2%  a  
  2π:  3 lx a   FOV:  3 lx a   -2%  a  
  2π:   0.422 a   FOV:  0.422 a   0%  a   
   
  
  
 
 
 
 
 Code 
       gtsave (Tabellendaten,  "Results/Table_TUB_BAuA_Studie_geneigt.png" ,  zoom =   4 ) 
    
    #Putting data to the Meta-Frame  
   Meta  <-   rbind (Meta,  
                  cbind ( Projekt =   "TUB-BAuA Studie" , Params))        
 
 
 
 
 TUB-BAuA Lab-Study (vertical) 
 
 
 Code 
       #Setting up the Information and Paths  
   Pfad  <-   "Data/TUB-BAuA/vertikal"  
   Pfad_Bild  <-   "Pictures/TUB-BAuA/vertikal"  
   Pfad_Spec  <-   "Results/TUB-BAuA/vertikal"  
   Wellenlängenspalte  <-   "Name"  
   file_params  <-   paste0 (Pfad,  "/Parameter.csv" ) 
   file_Spektrum  <-   paste0 (Pfad,  "/Messdaten.csv" ) 
    
    #Import the spectral Data  
   Spektraldaten  <-   read.csv (file_Spektrum,  sep =   ";" ,  dec =   "," )  %>%   
      select ( 1  :  13 ) 
    
    #Import the visual and nonvisual quantities  
   Params  <-   read.csv (file_params,  sep =   ";" ,  dec =   "," )  %>%  
      select ( 1  :  5 )  %>%   filter ( !  str_detect (Name,  "Mun" ))  %>%   
      separate (Name,  c ( "Name" ,  "SK" ),  sep =   "_" )  %>%   
      mutate ( SK =   recode (SK,  "SK"   =   "Blende" , 
                         .missing =   "Voll" ))  %>%   
      pivot_wider ( values_from =   c (E,MEDI, CCT),  names_from =  SK)  %>%   
      rowwise ()  %>%   
      mutate ( Bild =   image_file_path (Bild, Pfad_Bild), 
             Plot =   
               image_file_path ( str_c (Name,  ".png" )  %>%   
                                 str_replace ( " " ,  "." ), Pfad_Spec), 
             E_pct =  scales ::  percent ( -  1  +  round (E_Blende / E_Voll,  2 )), 
             MEDI_pct =  scales ::  percent ( -  1  +  round (MEDI_Blende / MEDI_Voll,  2 )) 
     )  %>%   
      select ( - CCT_Voll,  - CCT_Blende)  %>%   
      relocate (Bild, Plot)  %>%   
      rename ( "Szene"   =   "Name" ) %>%   
      arrange (Szene) 
    
    #Adjust Scene-Names  
   Bezeichnungen  <-  Params $ Szene  %>%   str_replace ( " " ,  "." ) 
    
    #Generating the Arguments for the spectral plots  
   Argumente  <-  
      list ( 
        Data =   list (Spektraldaten), 
        Bezeichnung_Wellenlaenge =   syms (Wellenlängenspalte), 
        Bezeichnung_Voll =   syms (Bezeichnungen), 
        Bezeichnung_Blende =   syms ( Blendennamen2 (Bezeichnungen)), 
        Ordner =   list ( paste0 (Pfad_Spec,  "/" )), 
        Bezeichnung =   as.list (Bezeichnungen) 
     ) 
    
    #Generating the spectral plots  
    pwalk (Argumente,Combined_Plot) 
    
    #Generating the table, displaying and saving it  
   Tabellendaten  <-   
    Table (Params,  NULL ) 
    
   Tabellendaten        
 
 

 
 
 
   
    
     
        
       rSPD 
       Name 
       Illuminance 
       MEDI 
       MDER 
     
   
   
        
   
 DIM 
  2π:  6 lx a   FOV:  6 lx a   -6%  a  
  2π:  3 lx a   FOV:  3 lx a   -5%  a  
  2π:   0.428 a   FOV:  0.431 a   1%  a   
        
   
 LS1 
  2π:  276 lx a   FOV:  254 lx a   -8%  a  
  2π:  164 lx a   FOV:  90 lx a   -45%  a  
  2π:   0.594 a   FOV:  0.353 a   -41%  a   
        
   
 LS2 
  2π:  178 lx a   FOV:  126 lx a   -29%  a  
  2π:  256 lx a   FOV:  238 lx a   -7%  a  
  2π:   1.438 a   FOV:  1.889 a   31%  a   
        
   
 LS3 
  2π:  181 lx a   FOV:  117 lx a   -35%  a  
  2π:  385 lx a   FOV:  111 lx a   -71%  a  
  2π:   2.127 a   FOV:  0.949 a   -55%  a   
   
  
  
 
 
 
 
 Code 
       gtsave (Tabellendaten,  "Results/Table_TUB_BAuA_Studie_vertikal.png" ,  zoom =   4 ) 
    
    #Putting data to the Meta-Frame  
   Meta  <-   rbind (Meta,  
                  cbind ( Projekt =   "TUB-BAuA Studie" , Params))        
 
 
 
 
 TU-Ilmenau Field-Study 
 
 
 Code 
       #Setting up the Information and Paths  
   Pfad  <-   "Data/TUI"  
   Pfad_Bild  <-   "Pictures/TUI"  
   Pfad_Spec  <-   "Results/TUI"  
   Wellenlängenspalte  <-   "Wellenlänge"  
   file_params  <-   paste0 (Pfad,  "/Parameter.csv" ) 
   file_Spektrum  <-   paste0 (Pfad,  "/Messungen.csv" ) 
    
    #Import the spectral Data  
   Spektraldaten  <-   read.csv (file_Spektrum,  sep =   ";" ,  dec =   "," ) 
    
    #Import the visual and nonvisual quantities  
   Params  <-   read.csv (file_params,  sep =   ";" ,  dec =   "," )  %>%  
      select ( 1  :  5 )  %>%   separate ( 
     Szene,  c ( "X" ,  "Y" ,  "APL" ,  "SK" ),  sep =   "_" ,  remove =   FALSE )  %>%   
      mutate ( SK =   recode (SK,  "SK"   =   "Blende" , 
                         .missing =   "Voll" ), 
             APL =   recode (APL,  "oAPL"   =   "nein" , 
                          "APL"   =   "ja" ), 
             Szene =   str_remove (Szene,  "_SK" ), 
             X =   str_replace (X,  "m" ,  "-" ), 
             Y =   str_replace (Y,  "m" ,  "-" ) 
            )  %>%   
      pivot_wider ( values_from =   c (E,MEDI, CCT),  names_from =  SK)  %>%   
          rowwise ()  %>%   
      mutate ( Bild =   image_file_path (Bild, Pfad_Bild), 
             Plot =   image_file_path ( str_c (Szene,  ".png" ), Pfad_Spec), 
             E_pct =  scales ::  percent ( -  1  +  round (E_Blende / E_Voll,  2 )), 
             MEDI_pct =  scales ::  percent ( -  1  +  round (MEDI_Blende / MEDI_Voll,  2 )) 
     )  %>%   
      select ( - CCT_Voll,  - CCT_Blende)  %>%   
      relocate (Y, Bild, Plot) 
    
    #Adjust Scene-Names  
   Bezeichnungen  <-  Params $ Szene 
    
    #Generating the Arguments for the spectral plots  
   Argumente  <-  
      list ( 
        Data =   list (Spektraldaten), 
        Bezeichnung_Wellenlaenge =   syms (Wellenlängenspalte), 
        Bezeichnung_Voll =   syms (Bezeichnungen), 
        Bezeichnung_Blende =   syms ( Blendennamen2 (Bezeichnungen)), 
        Ordner =   list ( paste0 (Pfad_Spec,  "/" )), 
        Bezeichnung =   as.list (Bezeichnungen) 
     ) 
    
    #Generating the spectral plots  
    pwalk (Argumente,Combined_Plot) 
    
    #Generating the tables, displaying and saving it  
   Tabellendaten  <-   Table (Params  %>%   filter (APL  ==   "nein" )  %>%   
                             mutate ( 
                               Y =   str_remove (Y,  "Y" )  %>%   str_c ( "°" ), 
                               X =   str_remove (X,  "X" )  %>%   str_c ( "°" ) 
                              ),  NULL )  %>%   
      cols_hide ( c (Szene, APL))  %>%   
      cols_label ( Y=  "Gaze Y" , 
                 X=  "Gaze X" )  %>%   
      cols_move (Y, X) 
    
   Tabellendaten        
 
 

 
 
 
   
    
     
        
       rSPD 
       Gaze X 
       Gaze Y 
       Illuminance 
       MEDI 
       MDER 
     
   
   
        — 
  
   
 0° 
 45° 
  2π:  532 lx a   FOV:  480 lx a   -10%  a  
  2π:  378 lx a   FOV:  339 lx a   -10%  a  
  2π:   0.709 a   FOV:  0.707 a   0%  a   
        — 
  
   
 0° 
 30° 
  2π:  437 lx a   FOV:  261 lx a   -40%  a  
  2π:  310 lx a   FOV:  184 lx a   -41%  a  
  2π:   0.709 a   FOV:  0.705 a   -1%  a   
        — 
  
   
 0° 
 15° 
  2π:  323 lx a   FOV:  98 lx a   -70%  a  
  2π:  229 lx a   FOV:  68 lx a   -70%  a  
  2π:   0.707 a   FOV:  0.695 a   -2%  a   
        
   
 0° 
 0° 
  2π:  195 lx a   FOV:  79 lx a   -59%  a  
  2π:  137 lx a   FOV:  55 lx a   -60%  a  
  2π:   0.703 a   FOV:  0.690 a   -2%  a   
        — 
  
   
 0° 
 -15° 
  2π:  112 lx a   FOV:  75 lx a   -33%  a  
  2π:  78 lx a   FOV:  52 lx a   -34%  a  
  2π:   0.698 a   FOV:  0.691 a   -1%  a   
        — 
  
   
 0° 
 -30° 
  2π:  92 lx a   FOV:  66 lx a   -29%  a  
  2π:  63 lx a   FOV:  45 lx a   -29%  a  
  2π:   0.689 a   FOV:  0.687 a   0%  a   
        — 
  
   
 0° 
 -45° 
  2π:  82 lx a   FOV:  55 lx a   -33%  a  
  2π:  56 lx a   FOV:  37 lx a   -34%  a  
  2π:   0.687 a   FOV:  0.680 a   -1%  a   
        — 
  
   
 -30° 
 0° 
  2π:  134 lx a   FOV:  87 lx a   -35%  a  
  2π:  94 lx a   FOV:  61 lx a   -35%  a  
  2π:   0.702 a   FOV:  0.699 a   0%  a   
        — 
  
   
 -15° 
 0° 
  2π:  154 lx a   FOV:  82 lx a   -47%  a  
  2π:  108 lx a   FOV:  57 lx a   -48%  a  
  2π:   0.703 a   FOV:  0.694 a   -1%  a   
        — 
  
   
 15° 
 0° 
  2π:  232 lx a   FOV:  84 lx a   -64%  a  
  2π:  164 lx a   FOV:  58 lx a   -65%  a  
  2π:   0.706 a   FOV:  0.693 a   -2%  a   
        — 
  
   
 30° 
 0° 
  2π:  280 lx a   FOV:  107 lx a   -62%  a  
  2π:  198 lx a   FOV:  75 lx a   -62%  a  
  2π:   0.707 a   FOV:  0.700 a   -1%  a   
        — 
  
   
 -30° 
 -15° 
  2π:  105 lx a   FOV:  73 lx a   -30%  a  
  2π:  73 lx a   FOV:  51 lx a   -30%  a  
  2π:   0.696 a   FOV:  0.695 a   0%  a   
        — 
  
   
 -15° 
 -15° 
  2π:  105 lx a   FOV:  70 lx a   -33%  a  
  2π:  73 lx a   FOV:  49 lx a   -33%  a  
  2π:   0.693 a   FOV:  0.692 a   0%  a   
        — 
  
   
 15° 
 -15° 
  2π:  141 lx a   FOV:  74 lx a   -48%  a  
  2π:  98 lx a   FOV:  51 lx a   -48%  a  
  2π:   0.699 a   FOV:  0.692 a   -1%  a   
        — 
  
   
 30° 
 -15° 
  2π:  160 lx a   FOV:  70 lx a   -56%  a  
  2π:  112 lx a   FOV:  48 lx a   -57%  a  
  2π:   0.701 a   FOV:  0.691 a   -1%  a   
   
  
  
 
 
 
 
 Code 
       gtsave (Tabellendaten,  "Results/Table_TU Ilmenau_ohne_APL.png" ,  zoom =   4 ) 
    
   Tabellendaten  <-   Table (Params  %>%   filter (APL  ==   "ja" )  %>%   
                             mutate ( 
                               Y =   str_remove (Y,  "Y" )  %>%   str_c ( "°" ), 
                               X =   str_remove (X,  "X" )  %>%   str_c ( "°" ) 
                              ),  NULL )  %>%   
      cols_hide ( c (Szene, APL))  %>%   
      cols_label ( Y=  "Gaze Y" , 
                 X=  "Gaze X" )  %>%   
      cols_move (Y, X) 
    
   Tabellendaten        
 
 

 
 
 
   
    
     
        
       rSPD 
       Gaze X 
       Gaze Y 
       Illuminance 
       MEDI 
       MDER 
     
   
   
        — 
  
   
 0° 
 45° 
  2π:  596 lx a   FOV:  564 lx a   -5%  a  
  2π:  415 lx a   FOV:  391 lx a   -6%  a  
  2π:   0.696 a   FOV:  0.693 a   0%  a   
        — 
  
   
 0° 
 30° 
  2π:  506 lx a   FOV:  337 lx a   -33%  a  
  2π:  350 lx a   FOV:  230 lx a   -34%  a  
  2π:   0.692 a   FOV:  0.681 a   -2%  a   
        — 
  
   
 0° 
 15° 
  2π:  391 lx a   FOV:  177 lx a   -55%  a  
  2π:  267 lx a   FOV:  116 lx a   -57%  a  
  2π:   0.684 a   FOV:  0.655 a   -4%  a   
        
   
 0° 
 0° 
  2π:  270 lx a   FOV:  160 lx a   -41%  a  
  2π:  182 lx a   FOV:  104 lx a   -43%  a  
  2π:   0.673 a   FOV:  0.647 a   -4%  a   
        — 
  
   
 0° 
 -15° 
  2π:  192 lx a   FOV:  150 lx a   -22%  a  
  2π:  126 lx a   FOV:  98 lx a   -22%  a  
  2π:   0.656 a   FOV:  0.653 a   -1%  a   
        — 
  
   
 0° 
 -30° 
  2π:  168 lx a   FOV:  135 lx a   -20%  a  
  2π:  110 lx a   FOV:  88 lx a   -20%  a  
  2π:   0.652 a   FOV:  0.651 a   0%  a   
        — 
  
   
 0° 
 -45° 
  2π:  147 lx a   FOV:  105 lx a   -28%  a  
  2π:  96 lx a   FOV:  68 lx a   -29%  a  
  2π:   0.654 a   FOV:  0.650 a   -1%  a   
        — 
  
   
 -30° 
 0° 
  2π:  203 lx a   FOV:  159 lx a   -21%  a  
  2π:  135 lx a   FOV:  105 lx a   -22%  a  
  2π:   0.665 a   FOV:  0.657 a   -1%  a   
        — 
  
   
 -15° 
 0° 
  2π:  236 lx a   FOV:  162 lx a   -31%  a  
  2π:  157 lx a   FOV:  105 lx a   -33%  a  
  2π:   0.666 a   FOV:  0.650 a   -2%  a   
        — 
  
   
 15° 
 0° 
  2π:  313 lx a   FOV:  163 lx a   -48%  a  
  2π:  212 lx a   FOV:  106 lx a   -50%  a  
  2π:   0.678 a   FOV:  0.651 a   -4%  a   
        — 
  
   
 30° 
 0° 
  2π:  335 lx a   FOV:  178 lx a   -47%  a  
  2π:  229 lx a   FOV:  118 lx a   -48%  a  
  2π:   0.684 a   FOV:  0.663 a   -3%  a   
        — 
  
   
 -30° 
 -15° 
  2π:  178 lx a   FOV:  138 lx a   -22%  a  
  2π:  117 lx a   FOV:  91 lx a   -22%  a  
  2π:   0.657 a   FOV:  0.655 a   0%  a   
        — 
  
   
 -15° 
 -15° 
  2π:  183 lx a   FOV:  144 lx a   -22%  a  
  2π:  120 lx a   FOV:  94 lx a   -22%  a  
  2π:   0.654 a   FOV:  0.652 a   0%  a   
        — 
  
   
 15° 
 -15° 
  2π:  213 lx a   FOV:  147 lx a   -31%  a  
  2π:  142 lx a   FOV:  96 lx a   -32%  a  
  2π:   0.665 a   FOV:  0.652 a   -2%  a   
        — 
  
   
 30° 
 -15° 
  2π:  222 lx a   FOV:  136 lx a   -39%  a  
  2π:  149 lx a   FOV:  88 lx a   -41%  a  
  2π:   0.672 a   FOV:  0.653 a   -3%  a   
   
  
  
 
 
 
 
 Code 
       gtsave (Tabellendaten,  "Results/Table_TU Ilmenau_mit_APL.png" ,  zoom =   4 ) 
    
   Tabellendaten  <-   Table (Params  %>%   
                             filter (Y  ==   "Y0" , X  ==   "X0" )  %>%   
                             mutate ( APL =   case_match (APL, 
                               "ja"   ~   "yes" , 
                               "nein"   ~   "no"  
                            ))  %>%   
                             arrange ( desc (APL)), 
          NULL )  %>%   
      cols_hide ( c (Szene, Y, X))  %>%   
      cols_label ( APL =   "Task Light" ) 
    
   Tabellendaten        
 
 

 
 
 
   
    
     
        
       rSPD 
       Task Light 
       Illuminance 
       MEDI 
       MDER 
     
   
   
        
   
 yes 
  2π:  270 lx a   FOV:  160 lx a   -41%  a  
  2π:  182 lx a   FOV:  104 lx a   -43%  a  
  2π:   0.673 a   FOV:  0.647 a   -4%  a   
        
   
 no 
  2π:  195 lx a   FOV:  79 lx a   -59%  a  
  2π:  137 lx a   FOV:  55 lx a   -60%  a  
  2π:   0.703 a   FOV:  0.690 a   -2%  a   
   
  
  
 
 
 
 
 Code 
       gtsave (Tabellendaten,  "Results/Table_TU Ilmenau_00.png" ,  zoom =   4 ) 
    
   Meta  <-   rbind (Meta, 
                  cbind ( Projekt =   "Montage- arbeitsplatz" , Params  %>%   select ( -  c (X,Y,APL))))        
 
 
 
 
 Gaze Direction (TU-Ilmenau) 
 
 
 Code 
       # Preparing the TU-Ilmenau Parameters for Gaze Direction Analysis  
   Params_numeric  <-  Params  %>%   mutate ( 
      across ( .cols=  c (X,Y),  ~   str_remove (.x,  "X|Y" )  %>%   as.numeric ()) 
   ) 
    
    # We need a Tibble of Gaze Directions in 15° steps  
   Points  <-   tibble ( Y=  seq ( -  45 , 45 , 15 ))  %>%   crossing ( X=   seq ( -  30 , 30 , 15 )) 
    
    # Plot creation  
   Params_numeric  %>%   filter (APL  ==   "ja" )  %>%   
      ggplot ( aes ( x= X,  y =  Y))  +  
      geom_point ( data= Points,  col =   "grey" )  +  
      geom_label ( aes ( label=  str_c (MEDI_Blende,  " lx  \n  " ,MEDI_pct),  
                     fill =  MEDI_Blende,  size =   -  1  + MEDI_Blende / MEDI_Voll))  +  
      theme_cowplot ( font_size =   8 ) +  
      scale_fill_viridis_c ( name =   "MEDI (lx)" ,  alpha =   0.5 ) +  
      scale_y_continuous ( breaks=  seq ( -  45 , 45 , 15 ),  
                         label =  scales ::  label_number ( suffix =   "°" ) )  +  
      scale_x_continuous ( breaks=  seq ( -  45 , 45 , 15 ),  
                         label =  scales ::  label_number ( suffix =   "°" ) )  +  
      scale_size_continuous ( name =   "Impact" ,  range =   c ( 4.5 , 2.5 ), 
                            label =  scales ::  label_percent ()) +  
      coord_cartesian ( xlim =   c ( -  32 , 32 ),  ylim =   c ( -  47 , 47 ),  clip =   "off" ) +  
      # labs(title = "Gaze-Direction in the vertical (y) and horizontal (x) direction greatly influence FOV (label size) and nonvisual stimulus (binned color)",  
           # subtitle = "") +  
      theme ( plot.title =   element_textbox_simple ( margin =   margin ( b=  2 )))        
 
 
   
 
 
 Code 
       ggsave ( "Results/Gaze-Direction.png" ,  height =   4 ,  width =   5 ,  units =   "in" )        
 
 
 
 
 Summary Analysis 
 
 
 Code 
       #Preserve the complete set of Measurements  
   Meta  %>%   write.csv ( file =   "Results/Meta.csv" ,  row.names =   FALSE ) 
    
    #Backup, should this chunk be used without the chunks above  
    # Meta <- read.csv("Results/Meta.csv")  
    
    #If Project-Names are used (which they are not), line-breaks are needed  
   Meta  <-  Meta  %>%   mutate ( Projekt=   str_replace (Projekt,  " " ,  "  \\\n  " )) 
    
    #We want a certain ordering of the projects  
   Projekte  <-   c ( "BAuA  \n  Studie" , 
                  "Homeoffice" ,  
                  "Industriehalle" , 
                  "Montage-  \n  arbeitsplatz" , 
                  "Lernraum" , 
                  "Industriehalle2" , 
                  "TUB-Halbk.  \n  Studie" , 
                  "TUB-BAuA  \n  Studie" ) 
    
   Meta  <-  Meta  %>%   mutate ( Projekt =   factor (Projekt,  levels =  (Projekte))) 
    
    #We need a summary dataframe for all the projects  
   Meta2  <-  Meta  %>%  
      group_by (Projekt)  %>%  
      summarize ( Median =   median ( 1  - MEDI_Blende / MEDI_Voll))  %>%  
      mutate ( Projektbild =   
               str_c ( "Pictures/Zusammenfassung/" , Projekt  %>%   
                       str_replace ( "  \\\n  " ,  "" ),  ".png" )) 
    
    #Create a Summary-Plot with project images on the x-Axis  
   Zusammenfassung  <-   
     Meta  %>%   
      ggplot ( aes ( x =  Projekt,  y =   1  - MEDI_Blende / MEDI_Voll))  +  
      stat_pointinterval ( col =   "red" , 
        point_interval =   "median_qi" , 
        slab_fill =   "black" , 
        slab_color =   "black" , 
        slab_size =   2 , 
        .width =   c ( 1 , 0.5 ), 
        interval_size_range =   c ( 0.75 ,  2 ), 
     ) +  
      stat_dots ( position =   position_nudge ( x=   0.11 ),  col =   "red" , 
        point_interval =   "median_qi" , 
        slab_fill =   "black" , 
        slab_color =   "black" , 
        binwidth =   0.02  
     ) +  
      geom_text ( data =  Meta2,  aes ( x= Projekt,  y= Median,  
                                  label =  scales ::  percent (Median,  accuracy =   1 )),  
                hjust =   1 ,  nudge_x =   -  0.1 ,  col =   "red" ) +  
      theme_cowplot () +  
      scale_x_discrete ( labels =   
                         str_c ( "<img src='" , Meta2 $ Projektbild,  "' width='50' /><br>" , 
                              LETTERS[ 1  :  length ( levels (Meta2 $ Projekt))])) +  
      scale_y_continuous ( labels =  scales ::  label_percent ( prefix =   "-" ))  +  
      labs ( x =   NULL ,  y =   "Impact of FOV occlusion on MEDI" ) +  
      coord_cartesian ( clip=  "off" ,  ylim =   c ( -  0.02 ,  NA )) +  
      theme ( axis.text.x =   element_markdown (), 
            axis.title.y =   element_text ( hjust =   1 )) 
      # labs(title = "Projektübersicht als Boxplot mit Einzel-Ergebnissen")  
    
   Zusammenfassung        
 
 
  Warning in png::readPNG(get_file(path), native = TRUE): Image uses 16-bit
channels but R native format only supports 8-bit, truncating LSB.  
 
 
   
 
 
 Code 
       #Export the Plot  
    ggsave ( 
      "Results/Summary.png" , Zusammenfassung,  height =   3.2 ,  width =   8 ,  units =   "in" )        
 
 
  Warning in png::readPNG(get_file(path), native = TRUE): Image uses 16-bit
channels but R native format only supports 8-bit, truncating LSB.  
 
 
 
 Summary table 
 
 
 Code 
       #Backup, should this chunk be used without the chunks above  
   Meta  <-   read.csv ( "Results/Meta.csv" ) 
    
    #We only wan´t certain scenarios in the summary table  
   Meta3  <-  Meta  %>%   
      slice ( c ( 21  :  19 ,  1  :  2 ,  4 ,  8 ,  10 ,  34 ,  49 ,  5  :  7 ,  12  :  14 ,  22 ,  28  :  30 ))  %>%   
      mutate ( Szene =   
              Szene  %>%   case_match ( 
                 "X0_Y0_oAPL"   ~   "without  \n  Task  \n  Lighting" , 
                 "X0_Y0_APL"   ~   "with Task  \n  Lighting" , 
                 .default =  Szene 
     )) 
    
    #repeated footnotes in the summary tables  
   Footnotes  <-     function (Data, rows) { 
     Data  %>%   
      tab_footnote ( 
        footnote =   "This viewing position equals the spectral measurement position" , 
        locations =   cells_body ( columns =  Bild,  rows =  rows), 
        placement =   "left"  
     )  %>%   
      tab_footnote ( 
        footnote =   "Relative Spectral Power Distribution (rSPD), Hemispheric (2π) Measurement in stark color, FOV in faded color" , 
        locations =   cells_column_labels (Plot))  %>%   
      tab_footnote ( 
        footnote =   "Melanopic Equivalent Daylight (D65) Illuminance (MEDI)" , 
        locations =   cells_column_labels (MEDI_Voll))  %>%   
      tab_footnote ( 
        footnote =   "Melanopic Daylight (D65) Efficacy Ratio (MDER)" , 
        locations =   cells_column_labels (MDER_Voll))  %>%   
      tab_footnote ( 
        footnote =   "2π = Hemispheric Measurements, FOV = Measurements witht the Field of View (FOV) occlusion" )  %>%   
      tab_options ( 
        footnotes.padding =   0  
     ) 
   } 
    
    #summary table 1: create, show, save  
   Postertabelle1  <-   
    Table (Meta3  %>%   slice_head ( n=  10 )  %>%   rownames_to_column (), NULL )  %>%  
      tab_row_group ( label =   "D. Industry Workplace" , 
                    rows =   9  :  10 )  %>%   
      tab_row_group ( label =   "C. Industry Field Study (Machine Workplace)" , 
                    rows =   7  :  8 )  %>%   
        tab_row_group ( label =   "B. Home Office Workplace" , 
                    rows =   4  :  6 )  %>%   
        tab_row_group ( label =   "A. Realistic Office Lab Study" , 
                    rows =   1  :  3 )  %>%   
      cols_hide ( "Projekt" )  %>%   
      tab_style ( style =   cell_text ( size =   "large" ), 
                locations =   list ( cells_row_groups (), cells_column_labels (), 
                                 cells_body (),  cells_footnotes (),  cells_stub ()))  %>%  
      tab_style ( style =   cell_text ( weight =   "bold" ),  
                locations =   cells_row_groups ())  %>%   
      cols_width ( ends_with ( "_Voll" )  ~  px ( 175 ), 
                Szene  ~   px ( 130 ), 
                 stub ()  ~   px ( 31 ))  %>%   
      Footnotes ( rows =   7  :  10 )  %>%   
      cols_align ( align =   "right" ,  columns =   ends_with ( "_Voll" )  |  Bild) 
    
   Postertabelle1        
 
 

 
 
 
   
     
     
     
     
     
     
     
   
   
    
     
        
        
       rSPD  1   
       Name 
       Illuminance 
       MEDI  2   
       MDER  3   
     
   
   
     
       A. Realistic Office Lab Study 
     
      1 
   
   
 warm low 
  2π:  296 lx a   FOV:  212 lx a   -28%  a  
  2π:  141 lx a   FOV:  101 lx a   -28%  a  
  2π:   0.476 a   FOV:  0.476 a   0%  a   
      2 
   
   
 cold low 
  2π:  298 lx a   FOV:  213 lx a   -29%  a  
  2π:  300 lx a   FOV:  214 lx a   -29%  a  
  2π:   1.007 a   FOV:  1.005 a   0%  a   
      3 
   
   
 cold bright 
  2π:  830 lx a   FOV:  592 lx a   -29%  a  
  2π:  838 lx a   FOV:  595 lx a   -29%  a  
  2π:   1.010 a   FOV:  1.005 a   0%  a   
     
       B. Home Office Workplace 
     
      4 
   
   
 Morning 
  2π:  450 lx a   FOV:  184 lx a   -59%  a  
  2π:  426 lx a   FOV:  171 lx a   -60%  a  
  2π:   0.947 a   FOV:  0.927 a   -2%  a   
      5 
   
   
 Daytime 
  2π:  390 lx a   FOV:  161 lx a   -59%  a  
  2π:  324 lx a   FOV:  131 lx a   -60%  a  
  2π:   0.831 a   FOV:  0.812 a   -2%  a   
      6 
   
   
 Night 
  2π:  139 lx a   FOV:  58 lx a   -58%  a  
  2π:  55 lx a   FOV:  23 lx a   -59%  a  
  2π:   0.397 a   FOV:  0.396 a   0%  a   
     
       C. Industry Field Study (Machine Workplace) 
     
      7 
   4     
   
 Morning 
  2π:  449 lx a   FOV:  433 lx a   -3%  a  
  2π:  412 lx a   FOV:  399 lx a   -3%  a  
  2π:   0.918 a   FOV:  0.920 a   0%  a   
      8 
   4     
   
 Night 
  2π:  156 lx a   FOV:  150 lx a   -4%  a  
  2π:  72 lx a   FOV:  70 lx a   -3%  a  
  2π:   0.463 a   FOV:  0.466 a   1%  a   
     
       D. Industry Workplace 
     
      9 
   4     
   
 without
Task
Lighting 
  2π:  195 lx a   FOV:  79 lx a   -59%  a  
  2π:  137 lx a   FOV:  55 lx a   -60%  a  
  2π:   0.703 a   FOV:  0.690 a   -2%  a   
      10 
   4     
   
 with Task
Lighting 
  2π:  270 lx a   FOV:  160 lx a   -41%  a  
  2π:  182 lx a   FOV:  104 lx a   -43%  a  
  2π:   0.673 a   FOV:  0.647 a   -4%  a   
   
  
   
     
        2π = Hemispheric Measurements, FOV = Measurements witht the Field of View (FOV) occlusion 
     
     
         1   Relative Spectral Power Distribution (rSPD), Hemispheric (2π) Measurement in stark color, FOV in faded color 
     
     
         2   Melanopic Equivalent Daylight (D65) Illuminance (MEDI) 
     
     
         3   Melanopic Daylight (D65) Efficacy Ratio (MDER) 
     
     
         4   This viewing position equals the spectral measurement position 
     
   
 
 
 
 
 Code 
       gtsave (Postertabelle1,  "Results/Table_Part1.png" ,  expand =   c ( 5 ,  20 ,  5 ,  0 )) 
    
     #summary table 2: create, show, save  
   Postertabelle2  <-   
    Table (Meta3  %>%   rownames_to_column ()  %>%   slice_tail ( n=  10 ), NULL )  %>%  
      tab_row_group ( label =   "H. Artificial Office Lab Study " , 
                    rows =   8  :  10 )  %>%     
        tab_row_group ( label =   "G. Halfdome Ganzfeld Lab Setup" , 
                    rows =   7 )  %>%   
        tab_row_group ( label =   "F. Industry Field Study (Packaging Workplace)" , 
                    rows =   4  :  6 )  %>%   
        tab_row_group ( label =   "E. Learning Space" , 
                    rows =   1  :  3 )  %>%   
      cols_hide ( "Projekt" )  %>%   
        tab_style ( style =   cell_text ( size =   "large" ), 
                locations =   list ( cells_row_groups (), cells_column_labels (), 
                                 cells_body (),  cells_footnotes (),  cells_stub ()))  %>%  
      tab_style ( style =   cell_text ( weight =   "bold" ),  
                locations =   cells_row_groups ())  %>%   
      cols_width ( ends_with ( "_Voll" )  ~  px ( 175 ), 
                Szene  ~   px ( 130 ))  %>%  
      Footnotes ( rows =   1  :  3 )  %>%   
      cols_align ( align =   "right" ,  columns =   ends_with ( "_Voll" )  |  Bild) 
    
    
   Postertabelle2        
 
 

 
 
 
   
     
     
     
     
     
     
     
   
   
    
     
        
        
       rSPD  1   
       Name 
       Illuminance 
       MEDI  2   
       MDER  3   
     
   
   
     
       E. Learning Space 
     
      11 
   4     
   
 Morning 
  2π:  261 lx a   FOV:  232 lx a   -11%  a  
  2π:  242 lx a   FOV:  215 lx a   -11%  a  
  2π:   0.927 a   FOV:  0.927 a   0%  a   
      12 
   4     
   
 Daytime 
  2π:  209 lx a   FOV:  164 lx a   -22%  a  
  2π:  128 lx a   FOV:  100 lx a   -22%  a  
  2π:   0.612 a   FOV:  0.610 a   0%  a   
      13 
   4     
   
 Evening 
  2π:  147 lx a   FOV:  87 lx a   -41%  a  
  2π:  54 lx a   FOV:  31 lx a   -43%  a  
  2π:   0.367 a   FOV:  0.356 a   -3%  a   
     
       F. Industry Field Study (Packaging Workplace) 
     
      14 
   
   
 Morning 
  2π:  460 lx a   FOV:  206 lx a   -55%  a  
  2π:  322 lx a   FOV:  151 lx a   -53%  a  
  2π:   0.702 a   FOV:  0.736 a   5%  a   
      15 
   
   
 Daytime 
  2π:  225 lx a   FOV:  154 lx a   -31%  a  
  2π:  158 lx a   FOV:  110 lx a   -31%  a  
  2π:   0.704 a   FOV:  0.712 a   1%  a   
      16 
   
   
 Night 
  2π:  153 lx a   FOV:  120 lx a   -22%  a  
  2π:  97 lx a   FOV:  79 lx a   -19%  a  
  2π:   0.633 a   FOV:  0.654 a   3%  a   
     
       G. Halfdome Ganzfeld Lab Setup 
     
      17 
   
   
 Halfdome 
  2π:  1,982 lx a   FOV:  1,829 lx a   -8%  a  
  2π:  2,323 lx a   FOV:  2,147 lx a   -8%  a  
  2π:   1.172 a   FOV:  1.174 a   0%  a   
     
       H. Artificial Office Lab Study  
     
      18 
   
   
 LS1 
  2π:  276 lx a   FOV:  254 lx a   -8%  a  
  2π:  164 lx a   FOV:  90 lx a   -45%  a  
  2π:   0.594 a   FOV:  0.353 a   -41%  a   
      19 
   
   
 LS2 
  2π:  178 lx a   FOV:  126 lx a   -29%  a  
  2π:  256 lx a   FOV:  238 lx a   -7%  a  
  2π:   1.438 a   FOV:  1.889 a   31%  a   
      20 
   
   
 LS3 
  2π:  181 lx a   FOV:  117 lx a   -35%  a  
  2π:  385 lx a   FOV:  111 lx a   -71%  a  
  2π:   2.127 a   FOV:  0.949 a   -55%  a   
   
  
   
     
        2π = Hemispheric Measurements, FOV = Measurements witht the Field of View (FOV) occlusion 
     
     
         1   Relative Spectral Power Distribution (rSPD), Hemispheric (2π) Measurement in stark color, FOV in faded color 
     
     
         2   Melanopic Equivalent Daylight (D65) Illuminance (MEDI) 
     
     
         3   Melanopic Daylight (D65) Efficacy Ratio (MDER) 
     
     
         4   This viewing position equals the spectral measurement position 
     
   
 
 
 
 
 Code 
       gtsave (Postertabelle2,  "Results/Table_Part2.png" ,  expand =   c ( 5 ,  20 ,  5 ,  0 )) 
    
    #put the summary tables together  
   Part1  <-   image_read ( path =   "Results/Table_Part1.png" ) 
   Part2  <-   image_read ( path =   "Results/Table_Part2.png" ) 
    
   Summary  <-   image_append ( c (Part1, Part2)) 
    
    image_write (Summary,  path =   "Results/Table_Summary.png" ,  format =   "png" )        
 
 
 
 
 FOV 
 
 
 Code 
       #Import the datasat  
   Polar_data  <-   read_csv ( "Data/wpd_datasets.csv" )        
 
 
  Rows: 57 Columns: 4
── Column specification ────────────────────────────────────────────────────────
Delimiter: ","
dbl (4): r.Binocular, A.Binocular, r.CIE, A.CIE

ℹ Use `spec()` to retrieve the full column specification for this data.
ℹ Specify the column types or set `show_col_types = FALSE` to quiet this message.  
 
 
 Code 
       #Tidy the dataat  
   Polar_data  <-  Polar_data  %>%   pivot_longer ( cols =   everything (), 
                                names_to =   c ( ".value" ,  "set" ), 
                                names_sep =   "[.]" ) 
    
   Polar_data  <-  Polar_data  %>%   mutate ( r =   ifelse (r  >   90 ,  90 , r), 
                                        set =   factor (set,  levels =   
                                                       c ( "Binocular" ,  "CIE" )), 
                                        set =   fct_recode (set, 
                                                         "CIE S026"   =   "CIE" ))  %>%   
      drop_na () 
    
    #create a dataset for binocular vision  
   Binokular2  <-  Polar_data  %>%   filter (set  ==   "Binocular" )  %>%   mutate ( 
      A =   case_when ( 
       A  <=   180   ~   90  - (A -90 ), 
        TRUE   ~   270  - (A -270 ) 
   ), 
    set =   factor (set,  levels =   c ( "Binocular" ,  "CIE" )))  %>%   
      add_row ( set =   "Binocular" ,  r =   90 ,  A =  0 )  %>%   
      add_row ( set =   "Binocular" ,  r =   90 ,  A =  360 ) 
    
    #create and save the plot for the FOV  
   Polar_data  %>%   
      ggplot ( aes ( x= A,  y= r)) +  
      geom_hline ( yintercept =   seq ( 10 ,  90 ,  by=   20 ),  col=  "grey90" ,  linewidth =   0.2 ) +  
      geom_area ( 
        data =  Binokular2,  outline.type =   "full" ,  alpha =   0.5 ,  fill =   "blue" ) +  
      geom_area ( outline.type =   "full" ,  alpha =   0.55 ,  fill =   "red" ) +  
      scale_y_continuous ( limits =   c ( 0 , 90 ), 
                         breaks =   seq ( 10 ,  90 ,  by=  20 ),  expand =   expansion (), 
                         labels =  scales ::  label_number ( suffix =   "°" )) +  
      scale_x_continuous ( limits =   c ( 0 , 360 ),  breaks =   seq ( 0 ,  330 ,  by =   30 ), 
                         labels =  scales ::  label_number ( suffix =   "°" )) +  
      coord_polar ( start =   -  1  /  2  * pi,  direction =   -  1 ) +  
      facet_grid ( ~ set) +  
      theme_minimal () +  
      theme ( axis.line.x =   element_blank (), 
            axis.line.y =   element_blank (), 
            panel.grid.minor  =   element_blank (), 
            panel.grid.major.y  =   element_blank (), 
            text =   element_text ( size =   15 ) 
           ) +  
      labs ( x =   NULL ,  y =   NULL )        
 
 
   
 
 
 Code 
       ggsave ( "Results/view.png" ,  height =   3.5 )        
 
 
  Saving 7 x 3.5 in image  
 
 
 
 
 Verification of FOV occlusion cutoff angles 
 
 
 Code 
      filepath  <-   "Data/Validation/rel_Illuminance.xlsx"  
    
   Validation_data  <-   read_xlsx (filepath) 
    
   Validation_data  <-  Validation_data  %>%   mutate ( Illuminance_rel =  Illuminance /  max (Illuminance), 
                               .by =  Type) 
    
   shaded_areas  <-   tibble ( 
      xmin =   0 , 
      ymin =   c ( -  90 ,  50 ), 
      xmax =   1 , 
      ymax =   c ( -  70 ,  90 ) 
   ) 
    
   Initial_plot  <-   function (Filter, Variable) { 
     Validation_data  %>%   filter ({{ Filter }} ==  0 )  %>%    
      ggplot ( aes ( x= {{ Variable }},  y= Illuminance_rel,  color= Type))  +  
       { 
          if ( substitute (Filter)  ==   sym ( "X" )){ 
                geom_rect ( data =  shaded_areas,  aes ( 
        ymin =  xmin,  xmin =  ymin,  ymax =  xmax,  xmax =  ymax),  fill =   "black" ,  
        alpha =  0.5 ,  color =   NA ,  inherit.aes =   FALSE ) 
         } 
         } +  
     Commons 
   } 
    
   Commons  <-   list ( 
      geom_point ( size =   2 ), 
      geom_path ( aes ( group= Run),  lwd =   1 ), 
      theme_cowplot (), 
      scale_color_viridis_d (), 
      geom_function ( fun =  \(x)  cos (x /  90  * (pi /  2 )),  inherit.aes =   FALSE , 
                  lwd =   1 ,  lty =   2 ), 
      scale_x_continuous ( breaks=  c ( seq ( -  90 , 90 , by=  20 ),  0 ),  
                       label =  scales ::  label_number ( suffix =   "°" ) ), 
      labs ( y=  "Relative Illuminance" ), 
      scale_y_continuous ( labels =  scales ::  label_percent ()) 
   ) 
    
   Caption  <-   "Validation of FOV occlusion cutoff angles in the vertical (A) and horizontal (B) direction, depending on Type of occlusion. 'Jeti' specifies the standard measurement, 'Preliminary' the preliminary FOV occlusion, and 'FOV occlusion' is eponymous. A dashed curve shows the idealized behavior of a cosine-adapted sensor. (A) Both FOV occlusion types have a similar cutoff angle. The non-zero beamwidth of the illuminant leads to some variance at the exact cutoff angles. (B) The FOV occlusion types behave almost identical in the horizontal direction and with minimal difference to the 2π measurement without any occlusion."  
    
   Plot1  <-   Initial_plot (X, Y)  +  
      guides ( color =   "none" ,  lwd =   "none" ) +  
      geom_vline ( aes ( xintercept =   0 ),  lty=  3 ) +  
      coord_flip () 
    
   Plot2  <-   Initial_plot (Y, X) 
    
   (Plot1  |  (Plot2  /   guide_area ()  +   
                plot_layout ( guides =   "collect" ,  heights =   c ( 3 , 1 ))))  +  
      plot_annotation ( caption =  Caption, 
                      tag_levels =   "A" , 
                      theme =   theme ( 
                        plot.caption =   element_textbox_simple () 
                     ))        
 
 
   
 
 
 Code 
       ggsave ( "Results/S3.png" ,  height =   4.75 )        
 
 
  Saving 7 x 4.75 in image  
 
 
 
 

 
 
 
   


  
