# Supplementary figures and images for: Influence of the Human Field of View on Visual and Non-Visual Quantities in Indoor Environments
Source: Clocks Sleep. 2023 Aug 29;5(3):476–98. doi: 10.3390/clockssleep5030032 (PMC10530223; doi:10.3390/clockssleep5030032)

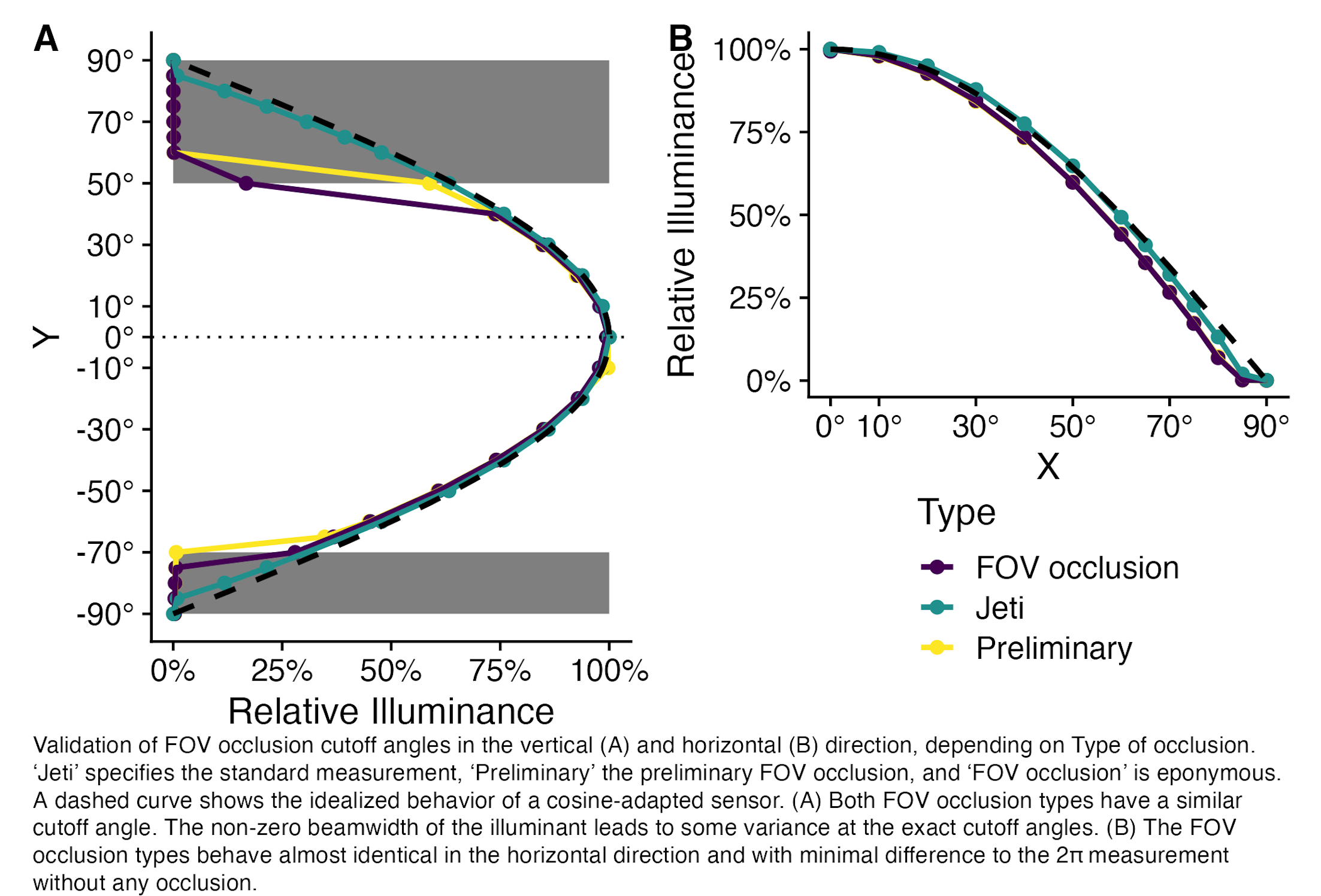

Supplement: Supplementary file 1 [file clockssleep-05-00032-s001.zip › 05-SI/FigureS3.png]

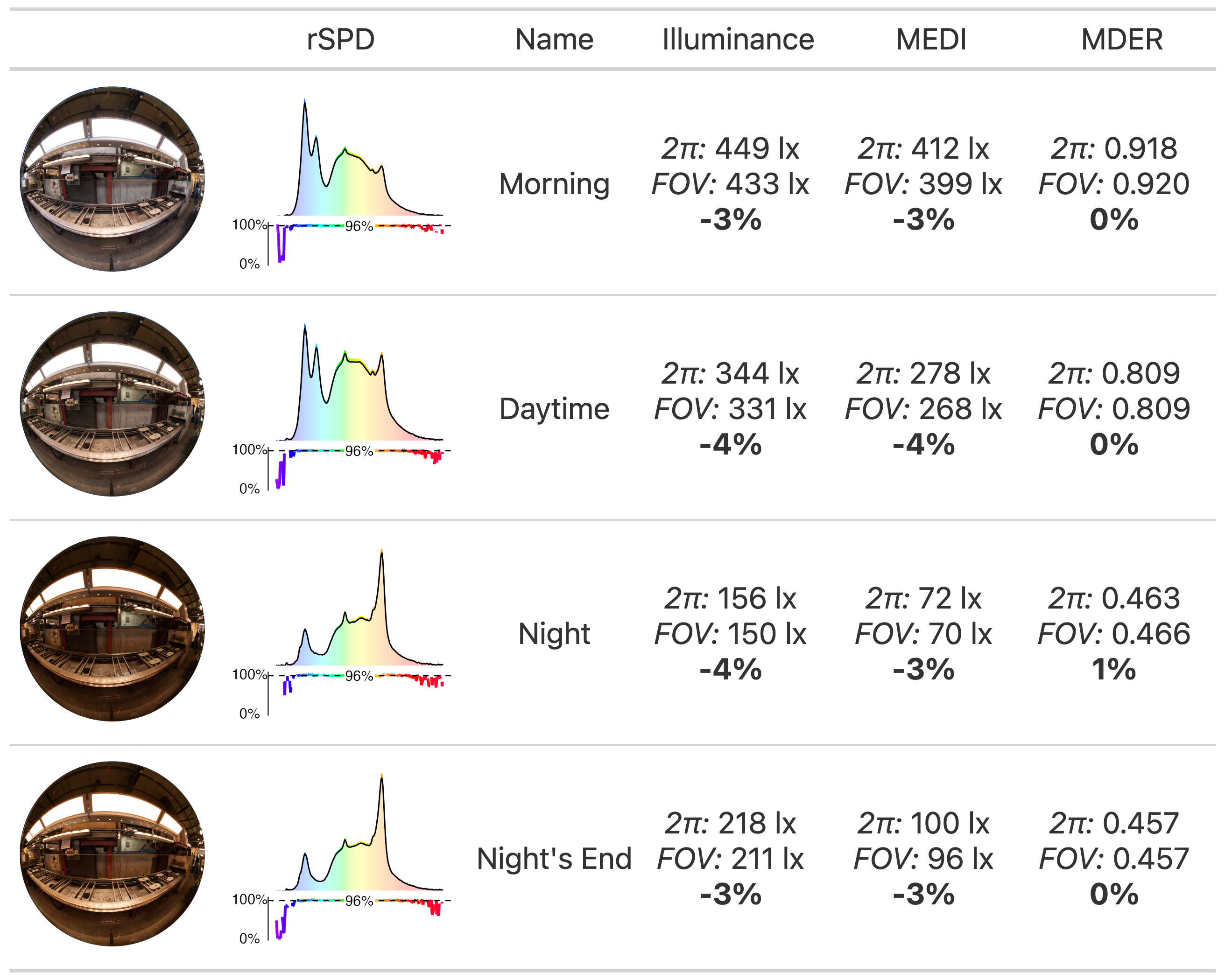

Supplement: Supplementary file 1 [file clockssleep-05-00032-s001.zip › 05-SI/SI1/ProjectC.png]

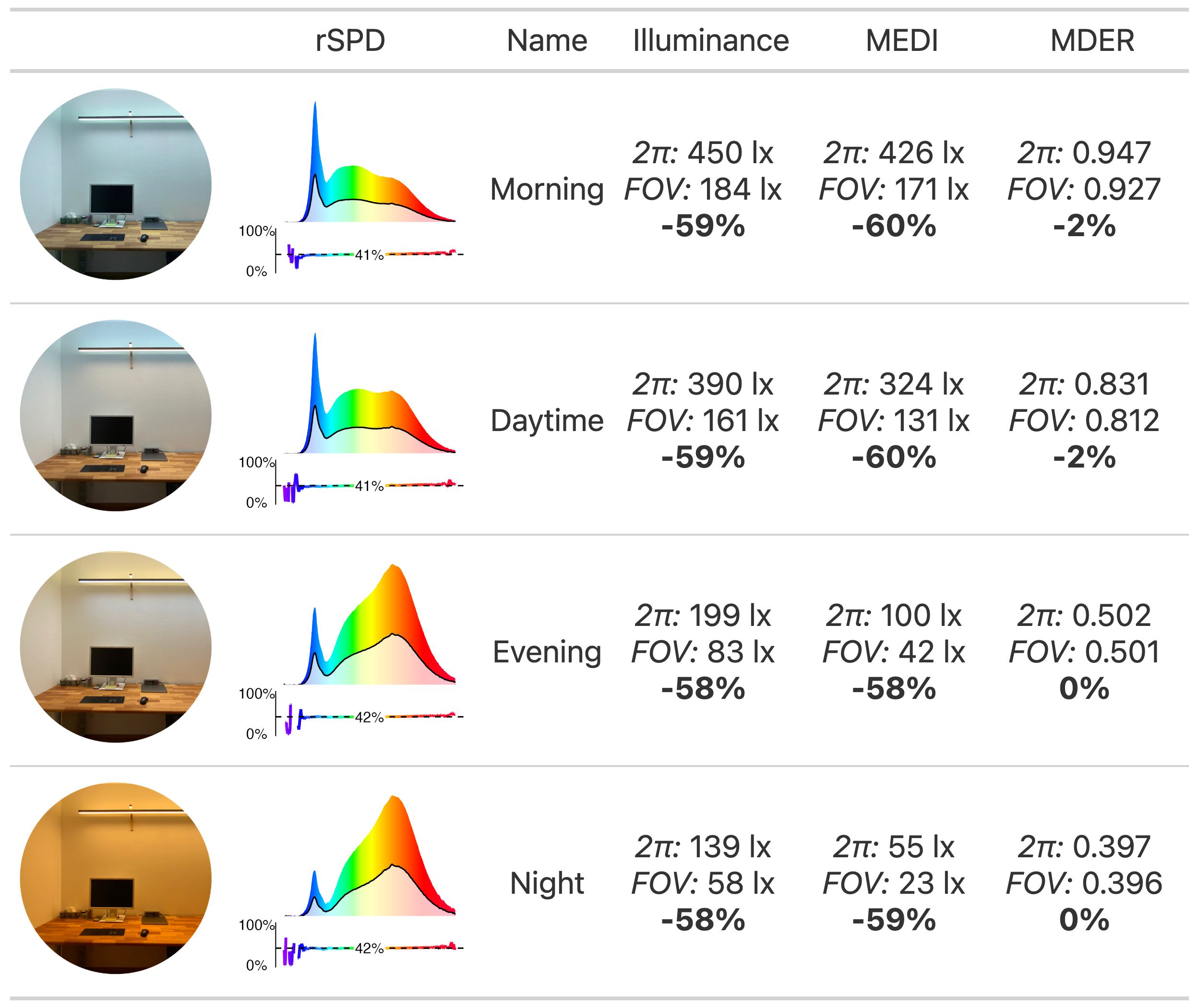

Supplement: Supplementary file 1 [file clockssleep-05-00032-s001.zip › 05-SI/SI1/ProjectB.png]

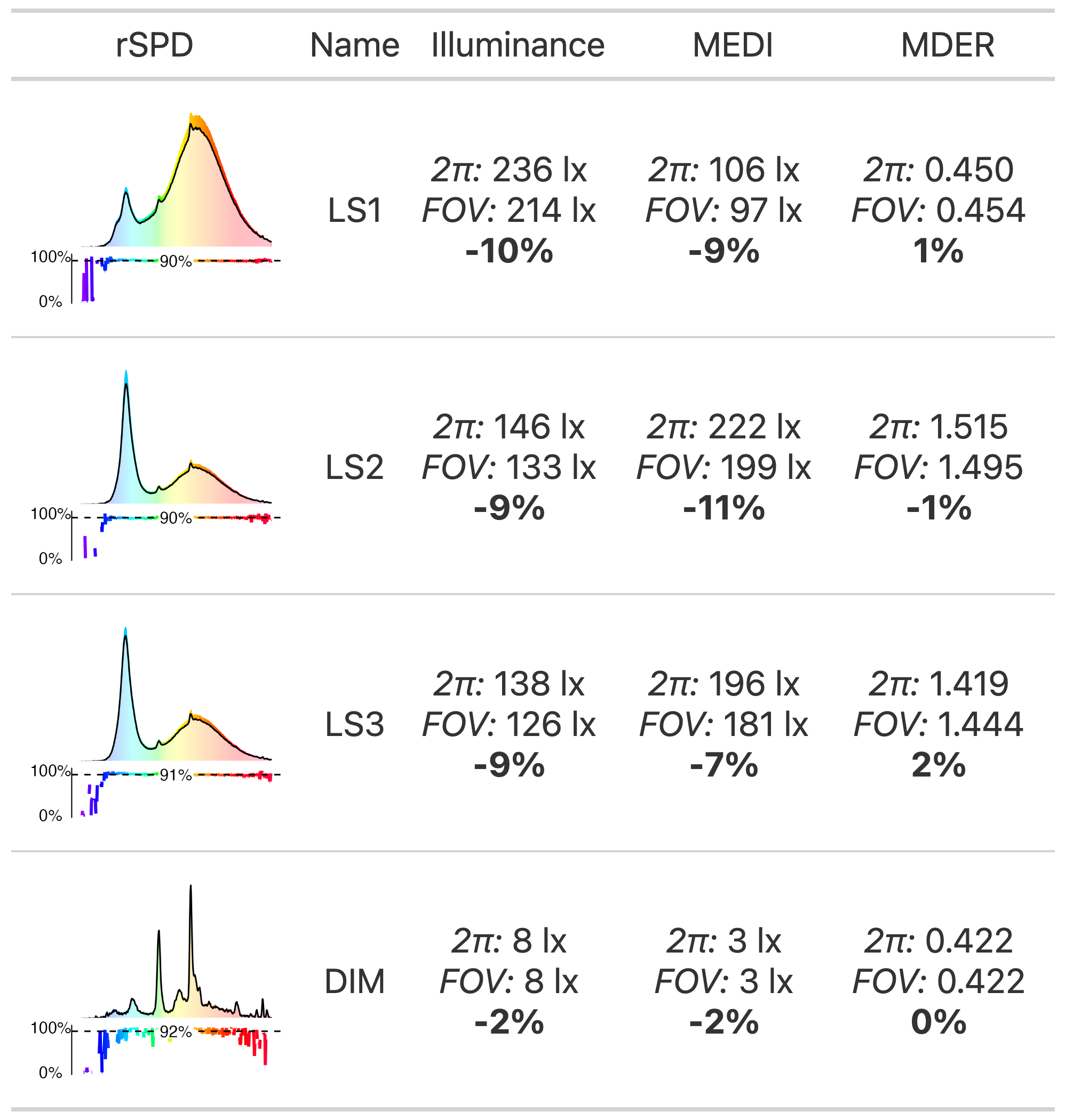

Supplement: Supplementary file 1 [file clockssleep-05-00032-s001.zip › 05-SI/SI1/ProjectH_tilt-20degree.png]

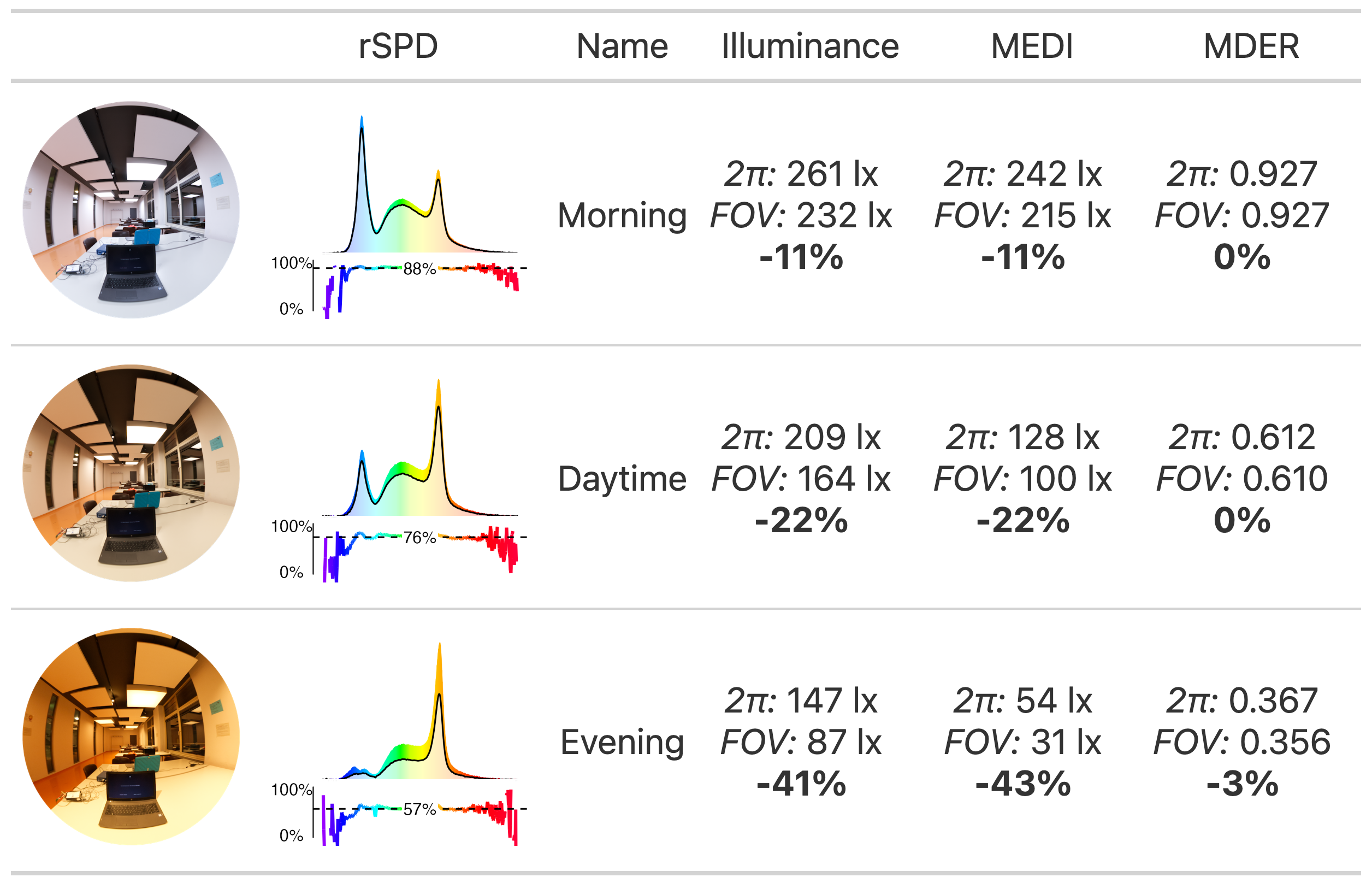

Supplement: Supplementary file 1 [file clockssleep-05-00032-s001.zip › 05-SI/SI1/ProjectE.png]

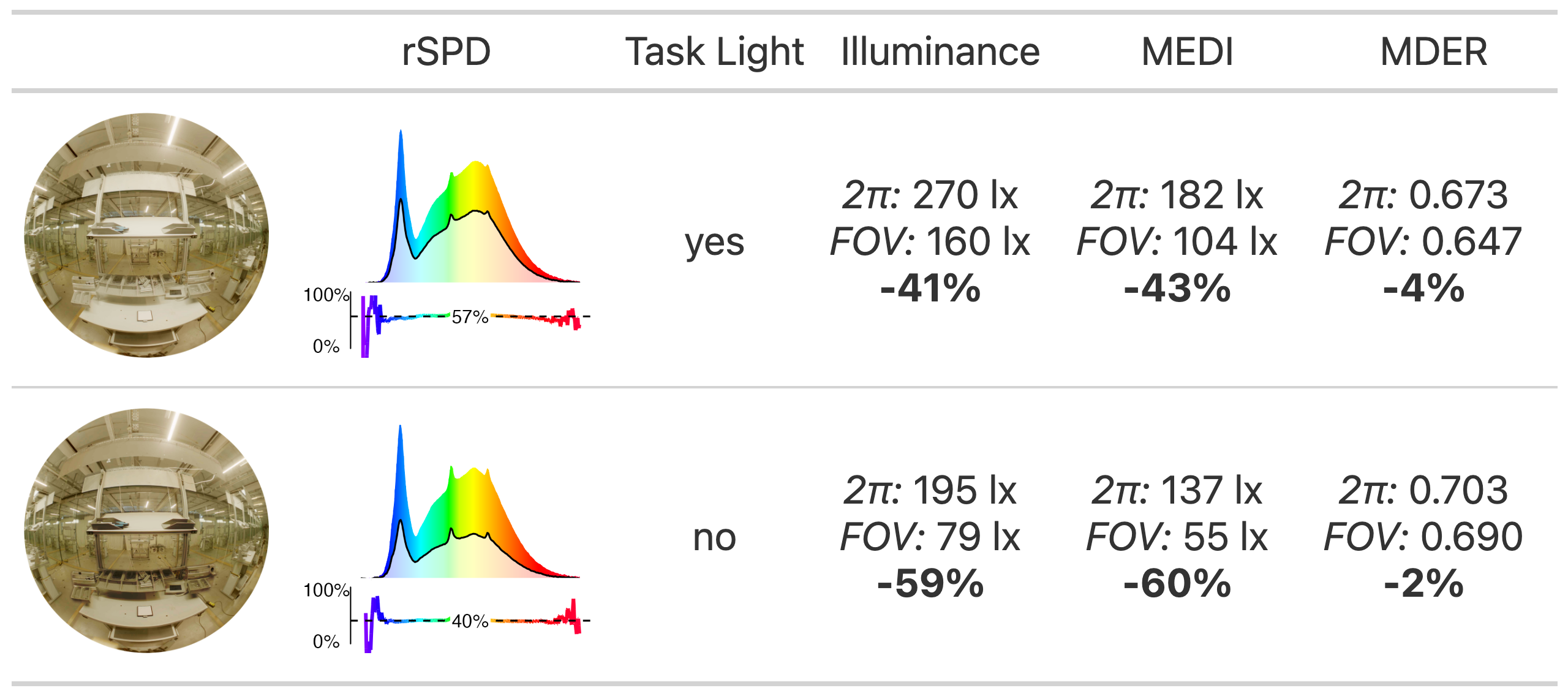

Supplement: Supplementary file 1 [file clockssleep-05-00032-s001.zip › 05-SI/SI1/ProjectD.png]

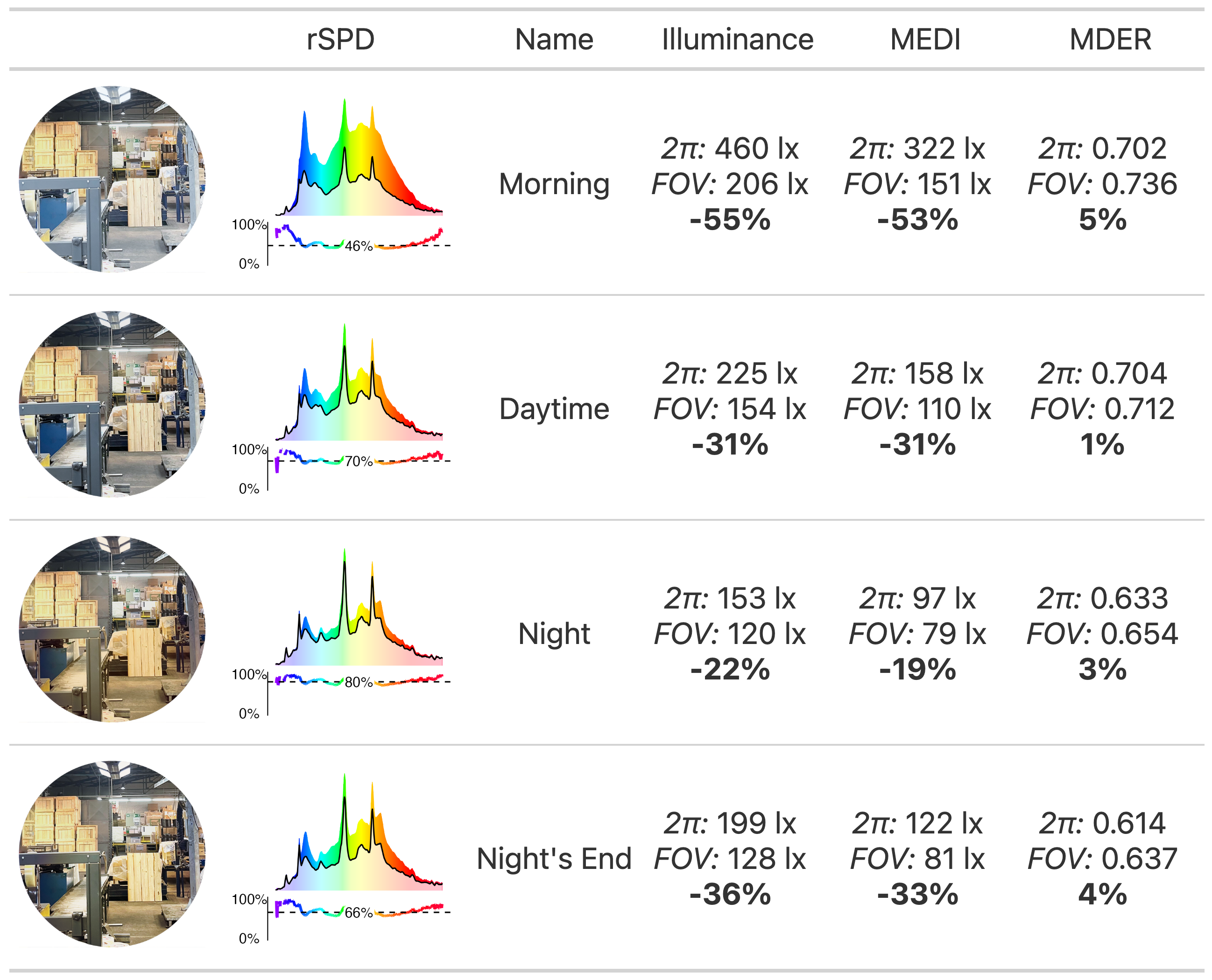

Supplement: Supplementary file 1 [file clockssleep-05-00032-s001.zip › 05-SI/SI1/ProjectF.png]

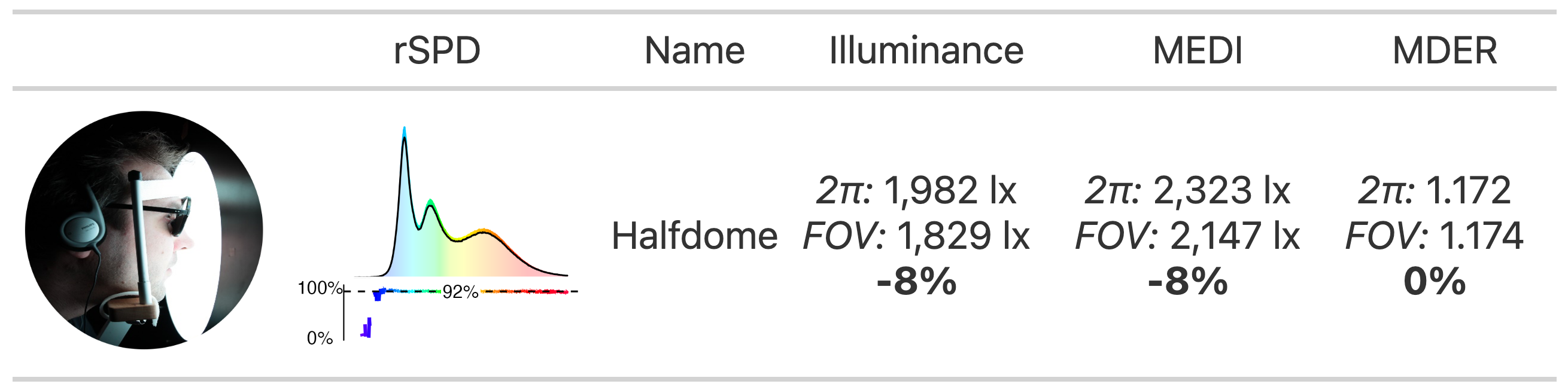

Supplement: Supplementary file 1 [file clockssleep-05-00032-s001.zip › 05-SI/SI1/ProjectG.png]

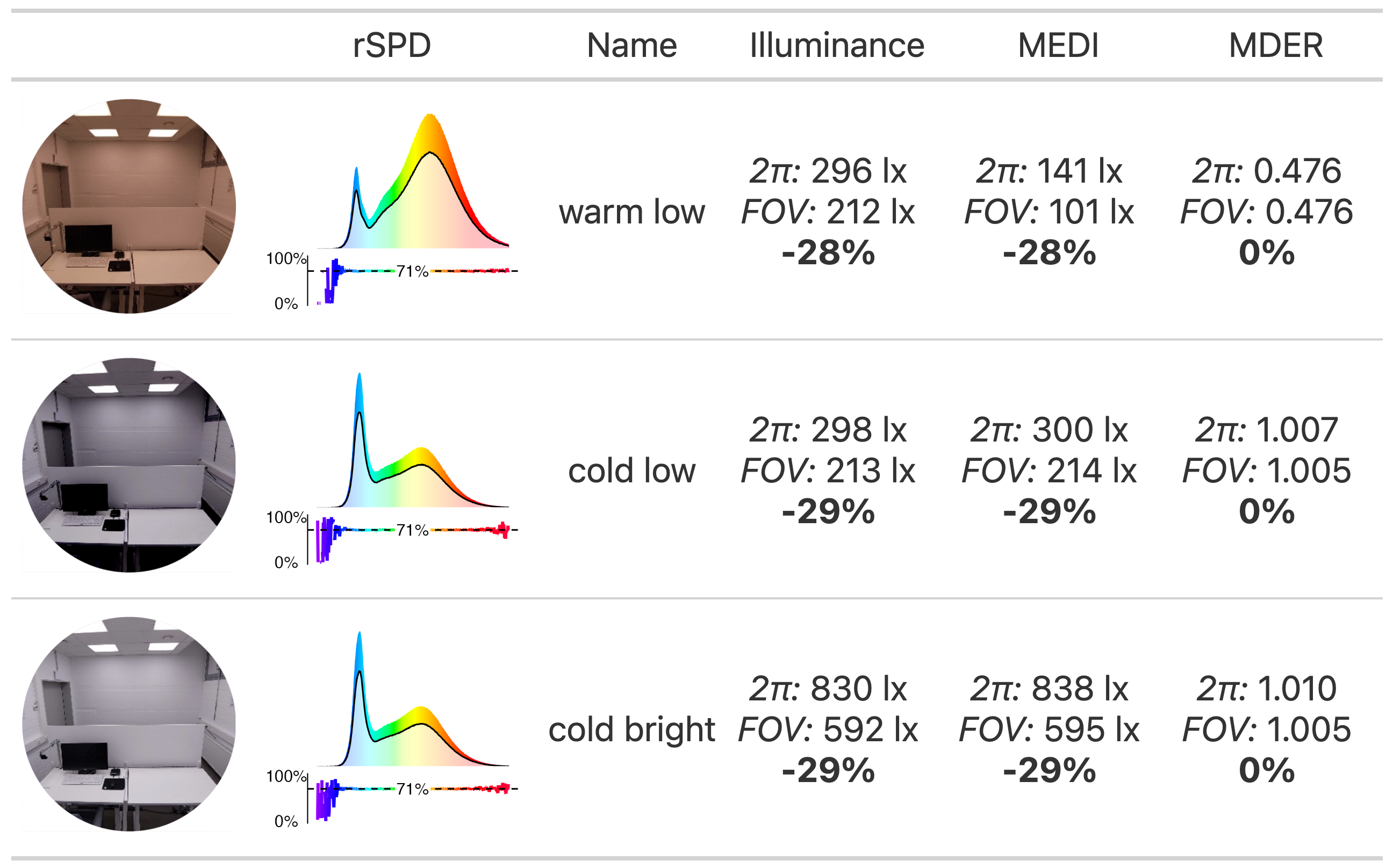

Supplement: Supplementary file 1 [file clockssleep-05-00032-s001.zip › 05-SI/SI1/ProjectA_tilt-0degree.png]

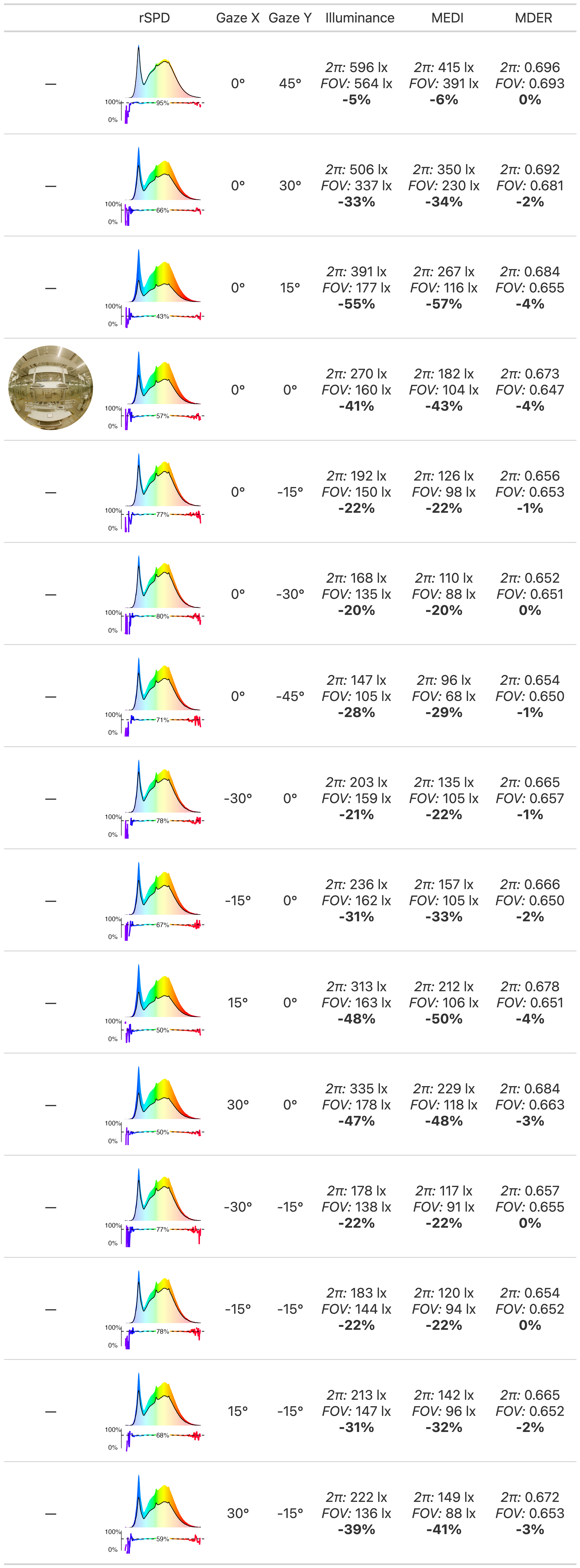

Supplement: Supplementary file 1 [file clockssleep-05-00032-s001.zip › 05-SI/SI1/ProjectD_all_directions_with_Tasklight.png]

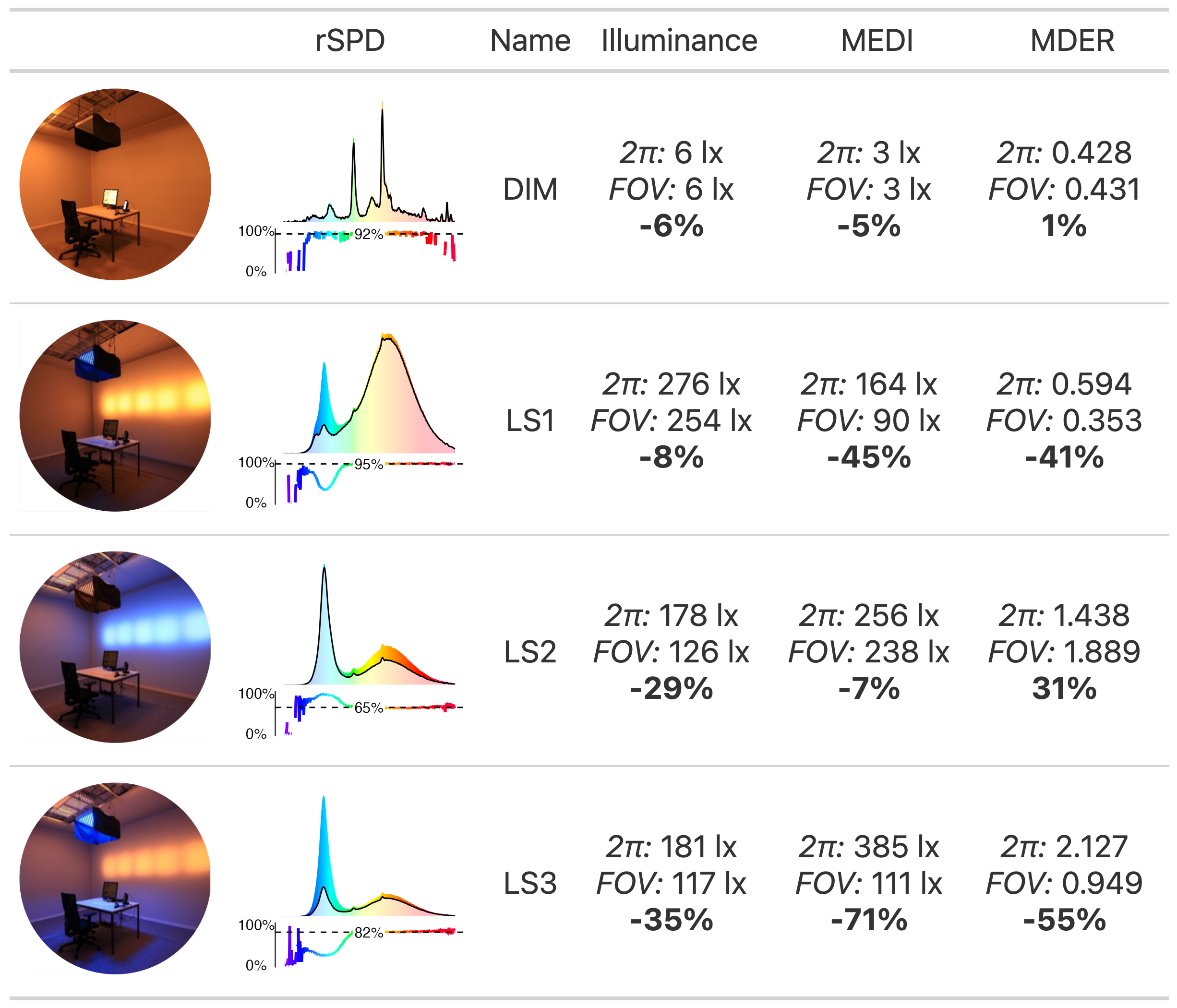

Supplement: Supplementary file 1 [file clockssleep-05-00032-s001.zip › 05-SI/SI1/ProjectH_tilt0degree.png]

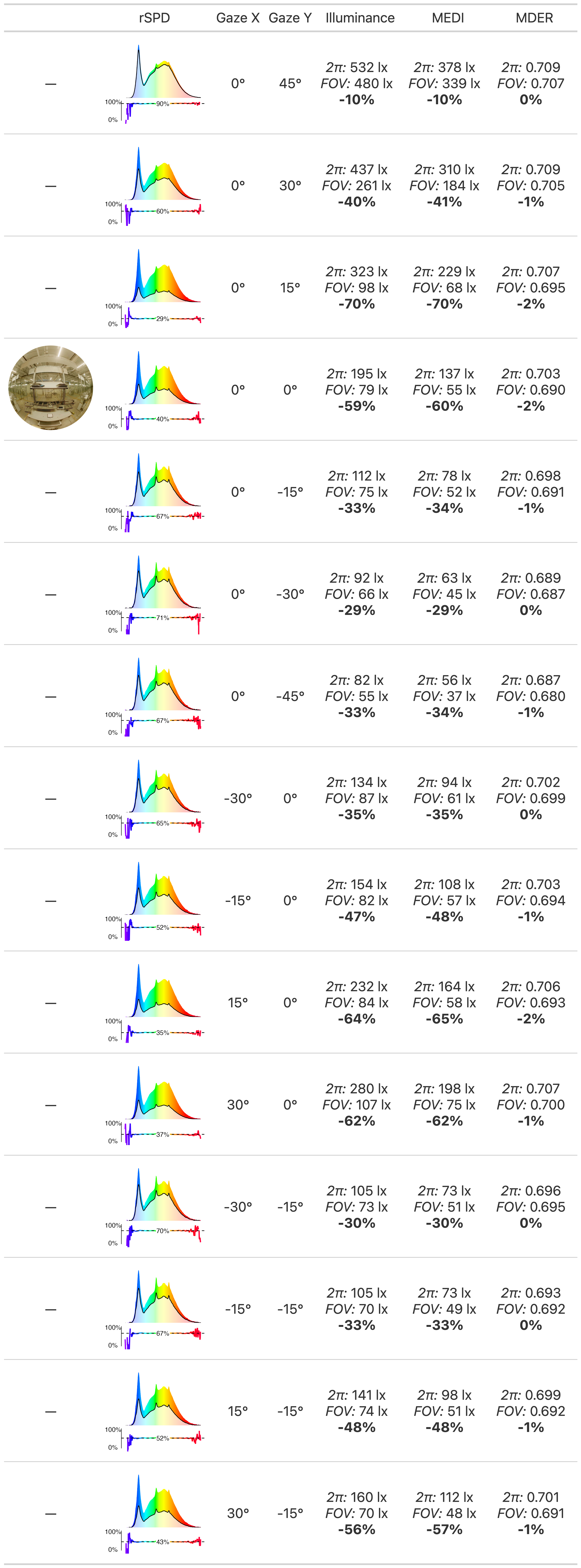

Supplement: Supplementary file 1 [file clockssleep-05-00032-s001.zip › 05-SI/SI1/ProjectD_all_directions_without_Tasklight.png]

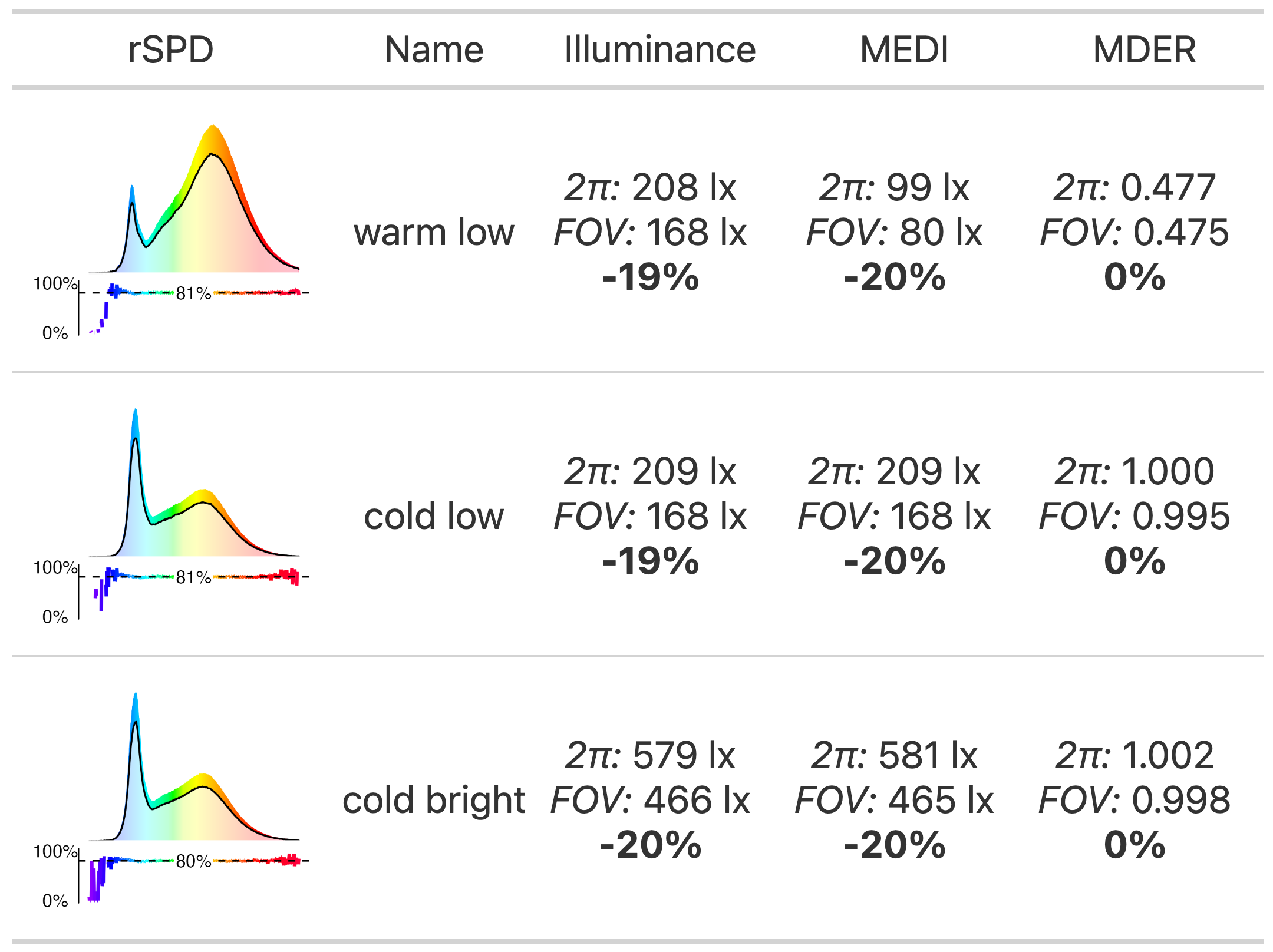

Supplement: Supplementary file 1 [file clockssleep-05-00032-s001.zip › 05-SI/SI1/ProjectA_tilt-20degree.png]
